# Niche divergence suggests ecophysiological differences between cryptic phytoplankton species

**DOI:** 10.1093/ismeco/ycag225

**Published:** 2026-08-04

**Authors:** Nicolas Romillac, Gianpaolo Zampicinini, Adriana Zingone, Diana Sarno

**Affiliations:** Department of Research Infrastructures for Marine Biological Resources, Stazione Zoologica Anton Dohrn, Villa Comunale, 80121 Naples, Italy; Department of Research Infrastructures for Marine Biological Resources, Stazione Zoologica Anton Dohrn, Villa Comunale, 80121 Naples, Italy; Department of Research Infrastructures for Marine Biological Resources, Stazione Zoologica Anton Dohrn, Villa Comunale, 80121 Naples, Italy; Department of Research Infrastructures for Marine Biological Resources, Stazione Zoologica Anton Dohrn, Villa Comunale, 80121 Naples, Italy; NBFC, National Biodiversity Future Center, Piazza Marina 61, 90133, Palermo, Italy

**Keywords:** cryptic species, LTER, metabarcoding, niche divergence, phytoplankton

## Abstract

Species-based ecology is fundamental to understanding and predicting the role of phytoplankton diversity in the production and biogeochemical cycles of marine ecosystems. However, microscopy fails to distinguish genetically distinct but morphologically identical (cryptic) or near-identical (pseudocryptic) species, which are lumped into species complexes under the assumption that they have similar ecological characteristics. To test this hypothesis, we analysed a 12-year V4-18S rDNA metabarcoding dataset (168 dates) from the Long-Term Ecological Research Site MareChiara (Gulf of Naples, Mediterranean Sea). Using Kernel Density Estimations and Outlying Mean Index niche analysis, we investigated the seasonality and ecology of 33 cryptic and pseudocryptic species or genetic variants, identified as amplicon sequence variants (ASVs). These belonged to eight species complexes, selected based on their abundance and frequency, within the diatoms *Leptocylindrus*, *Pseudo-nitzschia*, and *Chaetoceros*, the dinoflagellates *Azadinium* and *Heterocapsa*, and the chlorophyte *Micromonas*. Within each complex, ASVs exhibited distinct, annually recurrent seasonal dynamics, with peaks of the most abundant variants matching morphotype peaks for >60% of the timeline. Crucially, 75% of the sister ASVs rarely co-occurred (<30% of the samples), while their seasonality and ecological niches overlapped <60% in 72% and 56% of the couples, respectively. Divergence between species pairs suggests underrated ecophysiological differences and high adaptive potential within species complexes, highlighting the importance of identifying cryptic and pseudo-cryptic species. These findings demonstrate the power of long-term metabarcoding time-series in refining species-based ecological theories, while pointing to the need to uncover the molecular mechanisms driving phytoplankton phenology.

## Graphical Abstract

**Figure:**
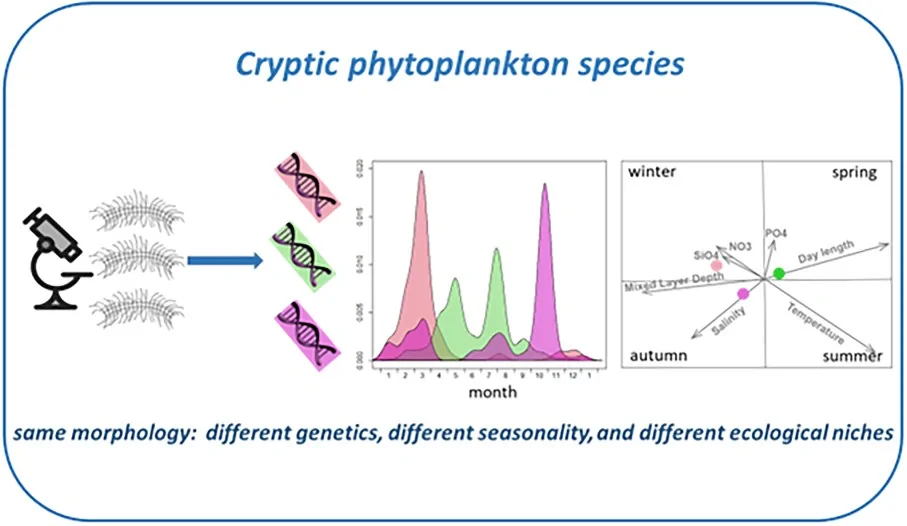

## Introduction

Phytoplankton diversity modulates essential processes in marine ecosystems such as primary productivity and biogeochemical cycling [1, 2]. Describing phytoplankton communities through a few functional or taxonomic groups might be sufficient to partly explain ecosystem functioning (e.g. [3]), but some important physiological and ecological traits are species-specific. Toxin production [4, 5], trophic habits [6], response to predation [7], symbiosis with N2-fixing cyanobacteria [8], and temperature and salinity preferences [9, 10] can strongly vary in closely related species and even between populations of the same species. However, characterizing phytoplankton communities at the species level requires high expertise and is often impossible in fixed material with traditional microscopy techniques. In addition, some species defined as cryptic (from the Greek ‘kryptos’ = hidden) are morphologically identical to others and can only be distinguished by their DNA sequences [11, 12], while others, defined as pseudocryptic, differ only in tiny ultrastructural details. Cryptic and pseudocryptic species are frequent and often co-occur in the same areas, but are poorly recognized and often lumped into species complexes [13].

Knowledge of the distribution and ecology of cryptic and pseudocryptic species was limited before the molecular age. The iconic marine diatom *Skeletonema costatum* was long thought to be ubiquitous and tolerant to a wide range of environmental conditions. However, the application of molecular approaches revealed that the same name encompassed several distinct species with different biogeographic and seasonal distributions [14]. Environmental DNA approaches have proven particularly powerful for facilitating investigations of planktonic communities at greater taxonomic resolution, providing information on rare species and on small and fragile taxa hardly identified in light microscopy [15, 16]. However, with a few exceptions (e.g. [17, 18]), metabarcoding studies have, thus far, primarily focused on the whole community, limiting the taxonomic analyses at most at the genus level (e.g. [19, 20]).

Given that cryptic microalgal species share morpho-functional traits (e.g. size, shape, colonial habit, and ultrastructural features) and are often closely related phylogenetically, they should also share ecological niches, i.e. thrive under similar environmental conditions [21]. In this case, to study biodiversity–ecosystem functioning relationships, they could be identified at the genus level or as species complexes without loss of information. This approach, which lumps taxa that have similar ecological requirements, is known as taxonomic sufficiency (TS) and is often applied in the field of bio-indication [22]. However, physiological and/or phenological traits not reflected in morphology can modulate individual fitness affecting growth, reproduction, and survival [23]. In terrestrial multicellular organisms, cryptic sister species may have different niches, support different ecological functions, and respond differently to climatic variations (e.g. [24, 25]). Resolving cryptic species’ distribution would then be necessary to improve the ecological knowledge of marine ecosystems.

Building upon prior research from the Mediterranean coastal Long-Term Ecological Research site *MareChiara* (LTER-MC, Gulf of Naples, GoN [26]), this study investigates the temporal dynamics of cryptic species, some of which were previously characterized using 1 or 3 years of molecular data [27–29]. We expand the analysis across a 12-year period, focusing on 5 complexes within the diatom genera *Leptocylindrus*, *Pseudo-nitzschia*, and *Chaetoceros*, alongside the small dinoflagellates *Azadinium* and *Heterocapsa* [4, 5] and the picoeukaryotic chlorophyte *Micromonas* [30]. By definition, the cryptic and pseudocryptic species groups examined in this study exhibit identical or nearly identical morphological traits, whereas their phylogenetic distances, reflecting their divergence times, vary from minimal in truly cryptic *Pseudo-nitzschia* species [31] to substantial in pseudocryptic *Leptocylindrus* [32], *Azadinium*, *Heterocapsa*, and *Micromonas*. Our primary objective was to test the hypothesis that cryptic or pseudocryptic species co-occur and occupy overlapping temporal and ecological niches, making their differentiation unnecessary. Conversely, the recognition of different seasonality and ecological requirements in sister species would provide new insights into species-based ecology, advancing our understanding of drivers of adaptation and evolution in phytoplankton communities.

## Material and methods

### Study site

The Long-Term Ecological Research MareChiara site (LTER-MC, 40°48.5′ N, 14°15′ E, 75 m depth) is located in the Gulf of Naples, two nautical miles from the coast (Fig. S1), in an area influenced by both coastal eutrophic waters and open sea oligotrophic waters [26, 33]. The site is characterized by pronounced interannual environmental variability while retaining a recurrent seasonal cycle for both abiotic variables and plankton [34, 35]. In surface waters, temperature ranges between 14 and 27°C and salinity between 36.2 and 38.2 across the year. The water column is stratified from April to October. Surface waters are depleted in nitrate and silicate from June to September, while nutrient peaks occur in March. Surface phytoplankton communities are dominated by diatoms of the genera *Chaetoceros*, *Leptocylindrus*, and *Pseudo-nitzschia*, which also include some of the species complexes here analysed, and generally present minima in winter and high abundance from late-winter to autumn, with irregular peaks in surface waters across the spring–summer periods. Phytoplankton populations are rather homogeneously distributed across the water column in mixed conditions, or concentrated in the upper layers during stratification, when waters below were scarcely populated and light limited [36].

### Sampling and environmental variables

In the LTER-MC dataset (1984 to date), we considered surface environmental data (0.5 m) from 168 dates coinciding with those of metabarcoding sampling (see below). To this aim, we used a data set obtained using a rosette sampler equipped with Niskin bottles and an SBE911 CTD (Sea-Bird Electronics). Temperature, salinity, and nutrient (nitrate, phosphate, silicate) concentrations data were obtained as previously described [37] and available at https://doi.org/10.5281/zenodo.7924734. Mixed layer depth was calculated according to [38].

### Selected species complexes and their light microscopy abundances

The eight species complexes analysed in this study were selected because they are known to include cryptic and pseudocryptic species that are identifiable by their diagnostic molecular signature, while their abundance across the time series allows sound statistical analyses. In the LTER dataset, there are other complexes with molecularly distinct but less abundant species (e.g. the dinoflagellate *Scrippsiella*). In general, the complexes selected cover a limited proportion of the whole phytoplankton population at LTER-MC in the dataset used for this study (e.g. 4.6% for *Leptocylindrus* and 15.1% for the three *Pseudo-nitzschia* complexes). Detailed taxonomic history and other information on the species complexes selected for this study are provided in the Supplementary Material S1.

Morphotypes of interest (referred to as ‘morphs’) from surface samples were enumerated in the Light Microscope (LM) at 400× magnification [39]. Subsamples of 1–100 ml fixed with neutralized formaldehyde (0.8%–1.6% final concentration) were let to settle and at least 200 cells of the most abundant taxa were counted. Abundance data are missing for *Micromonas*, and very limited for *Azadinium* and *Heterocapsa*, hardly identifiable in fixed material. *Leptocylindrus*-morph abundances were considered only starting from 2011, when *Leptocylindrus convexus* was identified as a separate species.

### DNA extraction and sequencing

The metabarcoding dataset consisted of 168 samples covering at least one monthly date per year, from July 2003 to December 2004 and from March 2010 to December 2019 (Supplementary File S1). Three litres of surface seawater were sampled as described in [40] and filtered onto 1.2 μm pore-size cellulose filters, except for the 2003–2004 samples, filtered on 3 μm pore-size filters. DNA was extracted with the DNeasy Plant Kit (Qiagen GmbH, Hilden, Germany). The hypervariable V4 region of the eukaryote SSU rRNA gene was amplified using the primers TAReuk454FWD1 and TAReukREV3 [41] modified as in [42]. Sequencing of 120 samples (A, 2x300 bp, paired end) was done using the Illumina MiSeq platform following the Illumina Nextera’s protocol (Illumina, San Diego, CA, USA) and subsequent modifications [43, 44] at the LGC Genomics GmbH facility (Berlin, Germany). Sequencing of 48 samples of the 2011–2013 period (B, 2x250 bp, paired end) was done at the CNR-IBIOM at the University of Bari, Italy (see [40]). The mean read number per sample was 121 373 in dataset A and 455 993 in dataset B.

### Amplicon sequence variants generation and taxonomic assignment

In both datasets, the quality of demultiplexed reads was assessed with FastQC [45]. Primer trimming of dataset A was performed by the sequencing service (LGC Genomics) using BBtools (http://jgi.doe.gov/data-and-tools/bbtools/), allowing up to three mismatches in the primer sequence. In dataset B, primers were trimmed using cutadapt v. 2.8 with adapter mismatch set at 0.25 [46]. Contig assembly, denoising, dereplication and chimera screening were performed using the dada2 library [47] in R, using the default parameters of the MiSeq tutorial (https://benjjneb.github.io/dada2/tutorial.html), except for the number of accepted mismatches in the overlap region (six in dataset A; eight in dataset B; default no mismatches). Amplicon sequence variants (ASVs) were taxonomically classified with the PR2 v4.14 database [48], supplemented with sequences obtained from strains cultivated from the GoN and submitted to GenBank, but not included in that version of PR2 (Supplementary File S1, column K). Non-protist ASVs (Metazoa, Fungi, and Streptophyta) were removed from the dataset.

The samples were rarefied 1000 times at a depth of 30 000 reads with the R package mirlyn [49] to have comparable ASV abundances in samples of different sequencing depths. The final ASV abundances were the average of the 1000 rarefied datasets, except for 3 samples that were slightly <30 000 reads and were not rarefied but left as they were. To assess the effect of the rarefaction depth, a sensitivity analysis was conducted on 88 samples (out of 168) with >100 000 reads. The samples were rarefied at 10 000, 30 000, 60 000, and 100 000 reads, and the relationship between the light microscopy and metabarcoding datasets (seasonal overlap and co-observation indexes, see below) for taxa quantified in both datasets was compared across subsamples.

Among the 161 ASVs matching a species of interest at >99% sequence identity (Supplementary File S1), we retained those present in at least 10 samples and with more than 100 reads for the ecological analysis. To refine taxonomic annotation, ASV sequences were blasted against the GenBank nucleotide database v2.13.0 to identify other identical reference sequences not included in PR2. Using the addfragment--keeplength function, ASV sequences were added to a MAFFT v7 alignment [50]—obtained with the L-INS-I method—of the whole-length 18S rDNA GenBank and PR2 reference sequences. Maximum likelihood trees were inferred using PhyML v3.0 [51] implemented in SeaView v5.0.5 [52], using a GTR + G + I substitution model and 1000 bootstrap replicates. ASVs resolved in the outgroup because of ill-identified reference sequences were eliminated from subsequent analysis. ASV annotation was further curated based on recent publications in the case of *Micromonas* [53] and *Pseudo-nitzschia* [31]. ASVs that could not be attributed with certainty (100%) to a species after those steps were discarded from the analysis, as they could be intra-genomic variants, a common case in protist genomes [27, 31].

### Seasonal overlap, co-occurrence, and niche overlap

The seasonal occurrence of a given taxon was expressed as the interquartile range (IQR) of the days of the year in which its ASVs were observed, i.e. the interval between the days corresponding to the 25^th^ and 75^th^ percentiles of the read distribution across the years. To explore the preferential environmental conditions of taxa occurrence, IQRs were also calculated for the physical and chemical variables concurrent with the ASVs’ records. Seasonality was then investigated in both ASV and LM data applying the kernel density estimation (KDE), a non-parametric, continuous, and smooth estimate of the population density probability along a gradient [54], in this case the months of the year, using the R package Ostats 0.1.1 [55]. With this approach, it was possible to calculate the seasonal overlap (SO) between two different taxa independently of their respective total abundance. To this aim, a common bandwidth parameter was calculated using the Scott formula [56] for all taxa belonging to the same cryptic group. To compare molecular and morphological results, seasonal overlap between KDEs of ASVs and of the corresponding morphs identified in LM (SoLM) was also calculated.

Ecological niches of cryptic species were analyzed using the Outlying Mean Index (OMI) [57] in the ade4 package [58]. This ordination approach represents the multidimensional space exploited by various taxa on a bi-dimensional plane, maximizing their separation. It characterizes each taxon by its tolerance, which is analogous to niche breadth, and marginality, i.e. the distance between the average conditions exploited by the species and the average conditions of the environment. The significance of the marginality was tested using a null model implemented via 100 random permutations under the null hypothesis that each taxon is indifferent to its environment [57]. The environmental variables used to define the bi-dimensional OMI plane were: daylength, temperature, salinity, mixed layer depth (MLD), and nutrient concentrations. Environmental variable and species abundance values (i.e. read numbers) were log-transformed prior to the analysis in order to downweight the highest abundances and extreme environmental values.

**Figure 1:**
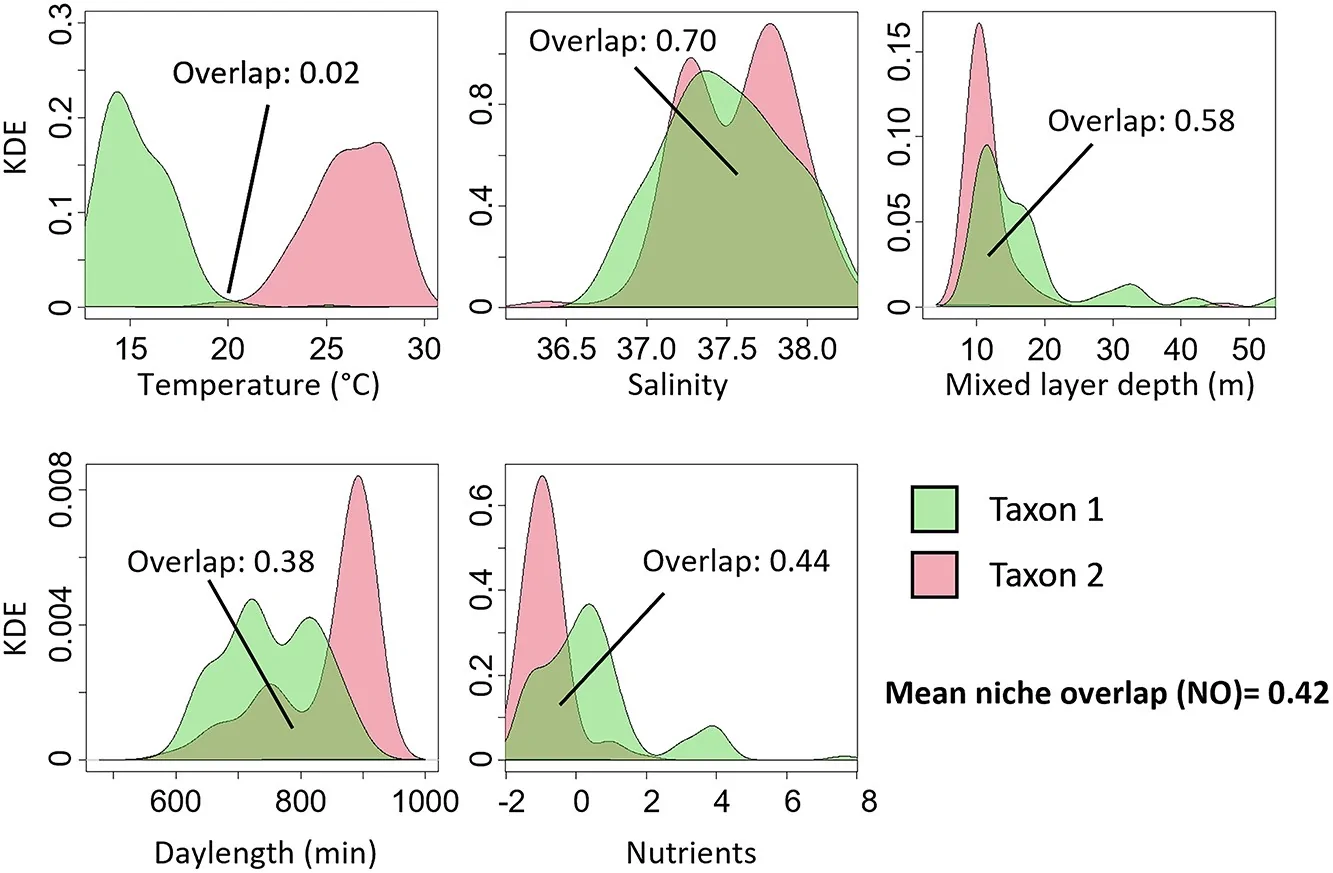
Theoretical example of the method used to calculate niche overlap between two species or intraspecific genetic variants (taxa). Taxa distributions along niche axes (environmental variables) are represented by kernel density probability estimation (KDE) curves [54], where the surface area under each KDE curve equals 1. For each variable, the overlap is calculated as the shared fraction of the total area bounded by both curves; these values are then averaged across all variables to obtain the mean niche overlap (NO) between the two taxa. Nutrient concentrations are reduced to a single dimension with PCA and represented as the first principal component (PC1) coordinates.

Niche overlaps (NOs) were calculated independently for each niche axis (environmental variables) retained in the OMI analysis using the KDE approach described above (Fig. 1). Due to strong correlation, nutrient concentrations were reduced to one dimension using principal component analysis (PCA). MLD was log-transformed as it is bound by 0, as recommended [59]. The mean niche overlap between two ASVs was calculated as the mean of overlaps on all niche axes [54].

Co-occurrence (CoOC) between taxa pairs was calculated as the number of samples containing both taxa divided by the number of samples with at least one of the two taxa [60]. The same approach was used to calculate co-observation (CoOB), that is, observation of a taxon in both metabarcoding and LM data from the same date.

Correlations between NO, SO, and CoOC were investigated with a PCA using package ade4.

To test if the co-occurrence, seasonality, and ecological niche overlaps within cryptic species pairs were statistically different from those of randomly picked species pairs, we created a comparison group [60]. CoOC, SO, and NO between each of our selected species and three randomly selected species pairs of the same taxonomic class were calculated. For each cryptic species pair, the CoOC (and, respectively, SO and NO) with their six randomly selected counterparts was averaged using the mean in order to obtain one comparison value for each cryptic pair. The comparison between CoOC, SO, and NO of the cryptic species pairs and the respective CoOC, SO, and NO of the randomly created species pairs was done using a paired Wilcoxon test.

Heatmaps of taxa abundance through the years were obtained with package IMIFA [61].

All analyses were done in R 4.1.3 [62].

## Results

### The metabarcoding dataset

Our rarefied dataset consisted of 69 945 eukaryotic ASVs, of which 15 378 assigned to Dinophyceae, 4777 to Bacillariophyceae, and 526 to Mamiellophyceae. Among them, 161 ASVs (227 907 reads) were assigned with >99% similarity to 42 reference sequences within the 8 marine phytoplankton complexes selected: the diatoms *Leptocylindrus*, *Pseudo-nitzschia delicatissima, Pseudo-nitzschia galaxiae, Pseudo-nitzschia pseudodelicatissima*, and *Chaetoceros curvisetus/pseudocurvisetus*; the dinoflagellates *Azadinium* and *Heterocapsa*, and the chlorophyte *Micromonas* (Supplementary File S1). The taxonomic history and other details of these complexes are provided in the Supplementary Material S1. Out of those 161 ASVs, 127 were discarded because of uncertain annotation, scarcely abundant or rare. The remnant 34 (215 764 reads) were retained for further analyses after refinement of their annotation through phylogenetic analysis (Table 1, Fig. S2). Two ASVs were lumped because they are known to be genomic variants of the same species, *Pseudo-nitzschia allochrona* (see below).

**Table 1:** List of ASVs attributed to the target cryptic taxa. The list includes all ASVs matching a reference sequence at 100%, or at >99% if abundant (>100 reads) and frequent (≥ 10 samples). The species names that were corrected manually are followed by the automated annotation in brackets. Reference sequence ID refers to the PR2 database, or to the GenBank nucleotide database when preceded by an asterisk. Taxa of uncertain taxonomy or too rare/infrequent were excluded from further analyses.

**ASV**	**ASV annotation**	**Reference sequence ID**	**Match (%)**	**Abundance** **(read *n*)**	**Occurrence** **(sample *n*)**	**Decision**
*Chaetoceros curvisetus*						
V4_MC2021_ASV_60_48d_122	*C. curvisetus* 1	MG972233.1.1691_U	100	4590	71	Retained
V4_MC2021_ASV_21_48d_113	*C. curvisetus* 2b	MG972240.1.1702_U	100	16 207	97	Retained
V4_MC2021_ASV_2039_48d_2656	*C. curvisetus* 3	MG972241.1.1787_U	100	140	23	Retained
V4_MC2021_ASV_186_48d_390	*C. pseudocurvisetus*	MG972305.1.1694_U	100	2822	87	Retained
*Leptocylindrus*						
V4_MC2021_ASV_4_48d_27	*L. aporus*	KC814810.1.1751_U	100	88 079	156	Retained
V4_MC2021_ASV_2041	*L. aporus*	KC814810.1.1751_U	100	79	1	Excluded^e^
V4_MC2021_ASV_46_48d_105	*L. convexus*	KC814811.1.1731_U	100	11 951	110	Retained
V4_MC2021_ASV_28_48d_64	*L. danicus*	KC814808.1.1725_U	100	16 139	116	Retained
V4_MC2021_ASV_674_48d_675	*L. hargravesii (L. danicus* str 23*)*^a^	KJ671698.1.702_gz_U	99.7	726	43	Retained
V4_MC2021_ASV_5436_48d_3656	*L. danicus* str 23	KJ671698.1.702_gz_U	100	29	9	Excluded^e^
*Pseudo-nitzschia delicatissima*						
V4_MC2021_ASV_268_48d_221	*P. allochrona*	KJ608076.1.885_U	100	2714	49	Retained
V4_MC2021_ASV_1968_48d_984	*P. allochrona*	KJ608076.1.885_U	99.7	258	11	Excluded^f^
V4_MC2021_ASV_363_48d_191	*P. delicatissima*	KJ608075.1.887_U	100	1898	41	Retained
V4_MC2021_ASV_286_48d_290	*P. arenysensis/dolorosa (P. dolorosa)* ^b^	*OK103849.1	100	1966	36	Retained
V4_MC2021_ASV_561_48d_372	*P. allochrona (P. dolorosa)* ^b^	*OK103849.1	99.7	1153	23	Retained^g^
*Pseudo-nitzschia galaxiae*						
V4_MC2021_ASV_455_48d_239	*P. galaxiae* B571	*OK103850.1	100	1191	25	Retained
V4_MC2021_ASV_202_48d_262	*P. galaxiae* B606	KJ608078.1.887_U	100	3083	51	Retained
V4_MC2021_ASV_176_48d_46	*P. galaxiae* B617	KJ608079.1.887_U	100	6924	66	Retained
V4_MC2021_ASV_546_48d_1314	*P. galaxiae* B617	KJ608079.1.887_U	99.7	896	73	Excluded^f^
*Pseudo-nitzschia pseudodelicatissima*						
V4_MC2021_ASV_1897_48d_551	*P. calliantha*	*OK103848.1	100	413	24	Retained
V4_MC48d_ASV_2252	*P. cuspidata*	*KP708995.1	100	35	2	Excluded^e^
V4_MC2021_ASV_12130_48d_3107	*P. hasleana (P. calliantha)* ^b^	JN091716.1.1113_U	100	27	9	Excluded^e^
V4_MC2021_ASV_3282_48d_1138	*P. mannii (P. calliantha)* ^b^	JN091716.1.1113_U	99.5	170	10	Retained
V4_MC2021_ASV_614_48d_1034	*P. caciantha/linea (P. granii)* ^b^	JN934671.1.1622_U	100	417	18	Retained
V4_MC2021_ASV_1031_48d_1653	*P. pseudodelicatissima*	KJ608082.1.887_U	100	501	14	Retained
*Azadinium*						
V4_MC2021_ASV_1317_48d_540	*A. concinnum/caudatum (A. concinnum)* ^c^	KJ481829.1.1794_U	100	496	10	Retained
V4_MC2021_ASV_2016	*A. cuneatum*	KJ481822.1.1794_U	99.2	137	23	Excluded^f^
V4_MC2021_ASV_1640_48d_1386	*A. dexteroporum*	KR362889.1.1795_U	100	219	18	Retained
V4_MC2021_ASV_3402_48d_3418	*A. obesum*	GQ914935.1.1499_U	100	47	4	Excluded^e^
V4_MC2021_ASV_956_48d_560	*Azadinium* sp.	KJ759591.1.1801_U	100	589	30	Retained
*Heterocapsa*						
V4_MC2021_ASV_177_48d_146	*H. limii* (*H. niei/rotundata*)^a^	FJ914475.1.1522_U	100	4332	61	Retained
V4_MC2021_ASV_219_48d_117	Unknown (*H. niei/rotundata*)^a^	HM561125.1.921_U	100	4406	34	Excluded^h^
V4_MC2021_ASV_296_48d_450	*H. niei/iwataki* (*H. niei* NaD18)^a^	*ON660934.1	100	1811	46	Retained
V4_MC2021_ASV_2320_48d_902	*H. pygmaea*	AF274266.1.1731_U	100	309	11	Retained
V4_MC2021_ASV_367_48d_545	*H. rotundata*	DQ388464.1.1702_U	100	1431	23	Retained
V4_MC2021_ASV_117_48d_319	*Heterocapsa* sp.	EF492494.1.1792_U	99.5	4835	38	Excluded^f^
V4_MC2021_ASV_75_48d_68	*Heterocapsa* sp.	JX661031.1.579_U	100	11 468	79	Retained
V4_MC48d_ASV_1073	*Heterocapsa* sp.	JX661031.1.579_U	99.7	153	30	Excluded^f^
V4_MC2021_ASV_593_48d_334	*H. horiguchii* (*H. triquetra*)^a^	FJ914432.1.1485_U	100	1195	21	Retained
*Micromonas*						
V4_MC2021_ASV_42_48d_36	*M. bravo* B1	KC488346.1.1634_U	100	15 594	75	Retained
V4_MC2021_ASV_99_48d_157	*M.* clade B4	AY425318.1.1734_U	100	5806	81	Retained
V4_MC2021_ASV_551_48d_651	*M.* clade B4	AY425318.1.1734_U	99.7	1012	28	Excluded^f^
V4_MC2021_ASV_345_48d_768	*M.* clade B5	KU743478.1.1776_U	100	1648	31	Retained
V4_MC2021_ASV_378_48d_516	*M. commoda* A1	KU743492.1.1777_U	100	1060	33	Retained
V4_MC48d_ASV_1139	*M. commoda* A1	KU743492.1.1777_U	99.7	107	17	Excluded^f^
V4_MC2021_ASV_409_48d_2593	*M. commoda* A2 (*M. pusilla*)^d^	KU244659.1.1703_U	100	450	14	Retained
V4_MC2021_ASV_83_48d_145	*M. bravo* B2 (*M. pusilla*)^d^	KU244665.1.1700_U	100	8272	96	Retained
V4_MC2021_ASV_1983	*M. bravo* B2 (*M. pusilla*)^d^	KU244665.1.1700_U	99.7	130	35	Excluded^f^

a Renamed after blasting in GenBank.

b Renamed based on ASV’s taxonomic attribution in [31].

c Renamed due to identical V4-18S rDNA shared by different species (see Fig. S2D).

d Renamed based on ASV’s taxonomic attribution in [53].

e Excluded due to low occurrence and abundance.

f Excluded due to unknown or unclear taxonomic attribution (including possible intragenomic variants).

g Retained and pooled with the other *P. allochrona* ASV because considered an intragenomic variant in [31].

h Excluded due to erroneous taxonomic attribution.

### Seasonality and ecology of cryptic taxa

Multiple statistical analyses and pairwise indices shed light on different aspects of the ecology and seasonal distribution of cryptic and pseudocryptic species and intraspecific variants in the species complexes addressed in this study (generally referred to as ‘taxa’).

**Figure 2:**
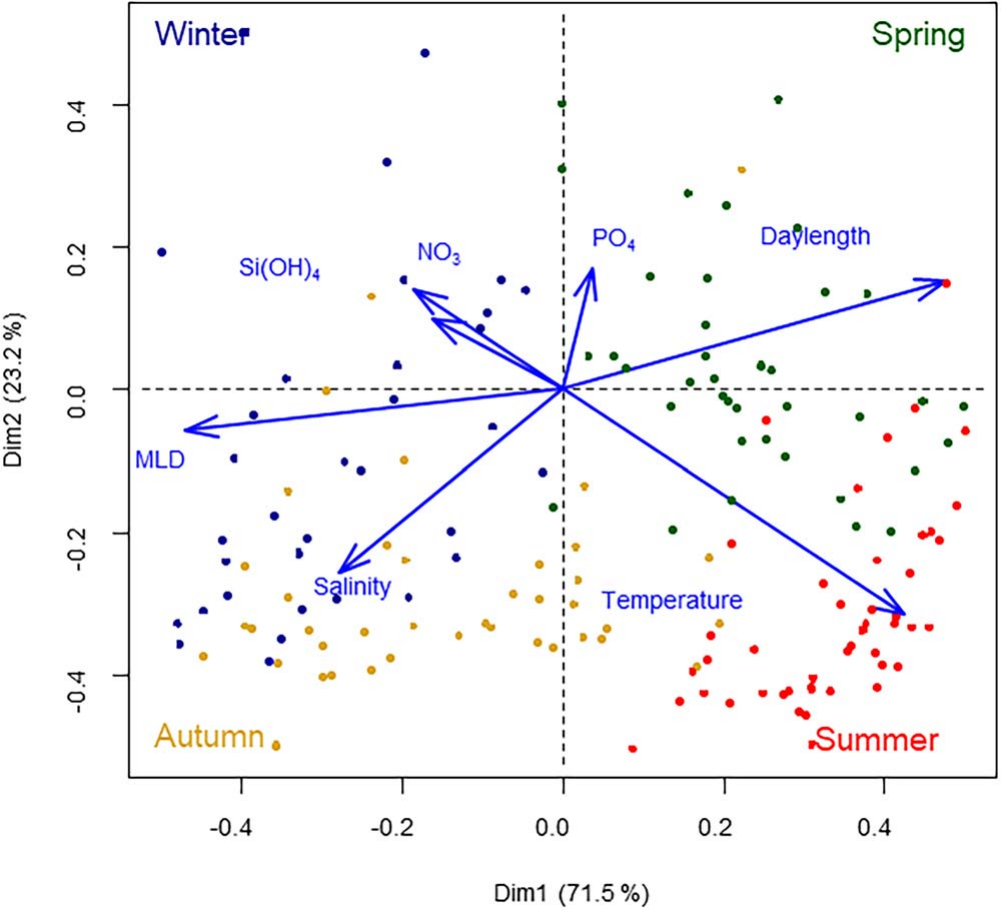
Bi-plot of the ‘common environmental space’, i.e. the multivariate ecological space exploited by the taxa, obtained from the OMI analysis. Points indicate samples, coloured according to the season of their collection date (Gregorian calendar). Arrows represent the environmental variables. Quadrants are arbitrarily identified as seasons based on the prevalence of the respective sampling dates. MLD, mixed layer depth; NO3, nitrate concentrations; PO4, phosphate concentrations; Si(OH)4, silicate concentrations. The niche parameters relative to the taxa examined are provided in Table S1.

**Figure 3:**
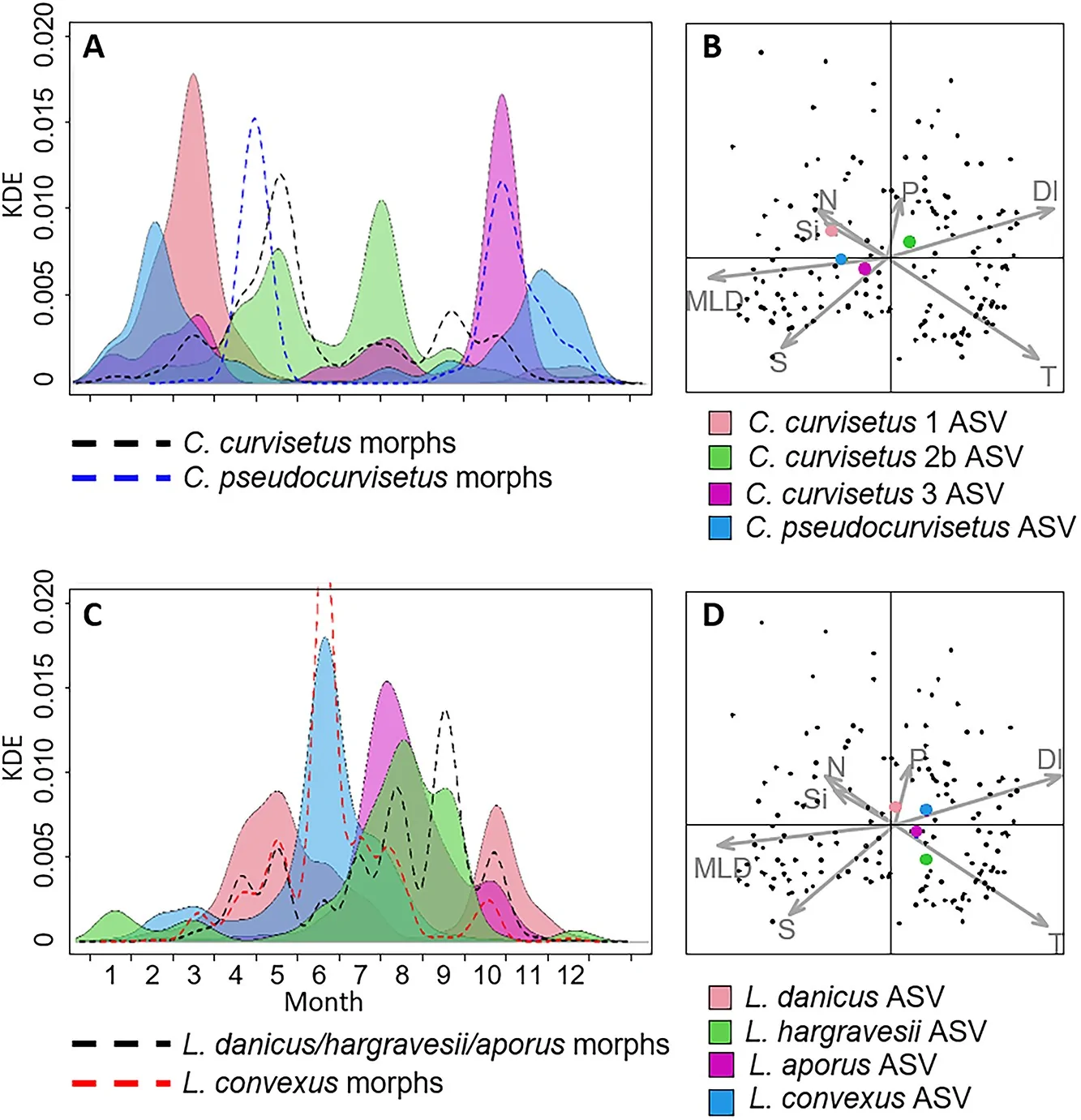
Seasonality, as KDE curves, and ecological niches of the *C. curvisetus* complex (A, B) and *Leptocylindrus* spp. (C, D). Dashed lines represent the KDE curves of morphological species from LM data. Ecological niches (B, D) are represented on the common environmental space defined by the first and second axes of the OMI analysis (Fig. 2). Niche positions are represented by coloured dots (see legend for colour code), with larger distances of the dots from the plane centre indicating more specialized niches. Arrows represent the environmental variables characterizing the ecological niche. T, temperature; S, salinity; MLD, mixed layer depth; N, nitrate concentration; P, phosphate concentration; Si, silicate concentration; Dl, daylength. See Fig. 2 for the canonical weights of the environmental variables and Table S1 for OMI parameters and niche statistical significance.

**Figure 4:**
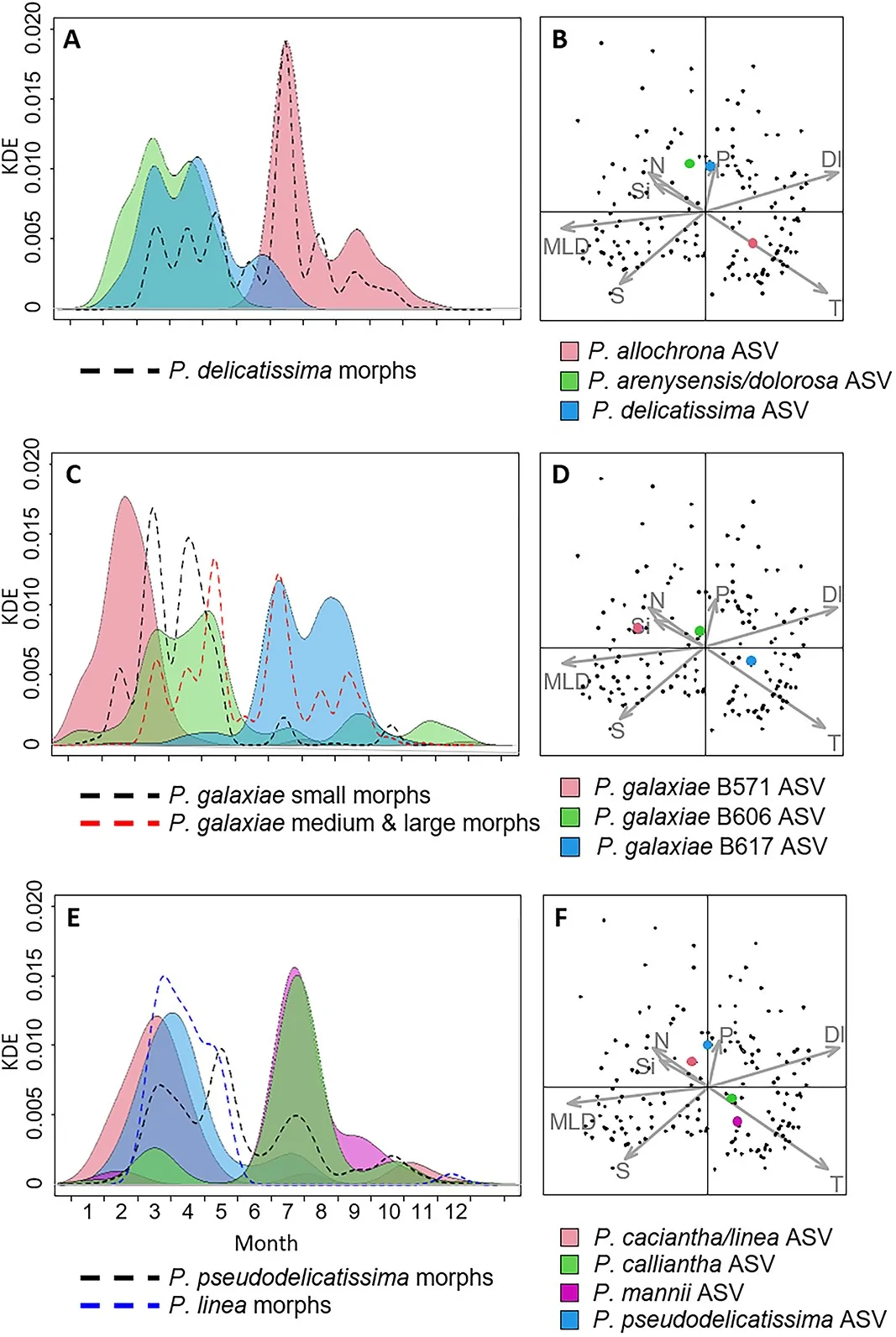
Seasonality, as KDE curves, and ecological niche of the *P. delicatissima* (A, B), *P. galaxiae* (C, D), and *P. pseudodelicatissima* complexes (E, F). Dashed lines represent the KDE curves of morphological species from LM data. Ecological niches (B, D, F) are represented on the common environmental space defined by the first and second axes of the OMI analysis (Fig. 2). Niche positions are represented by coloured dots (see legend for colour code), with larger distances of the dots from the plane centre indicating more specialized niches. Arrows represent the environmental variables characterizing the ecological niche. T, temperature; S, salinity; MLD, mixed layer depth; N, nitrate concentration; P, phosphate concentration; Si, silicate concentration; Dl, daylength. See Fig. 2 for the canonical weights of the environmental variables and Table S1 for OMI parameters and niche statistical significance.

**Figure 5:**
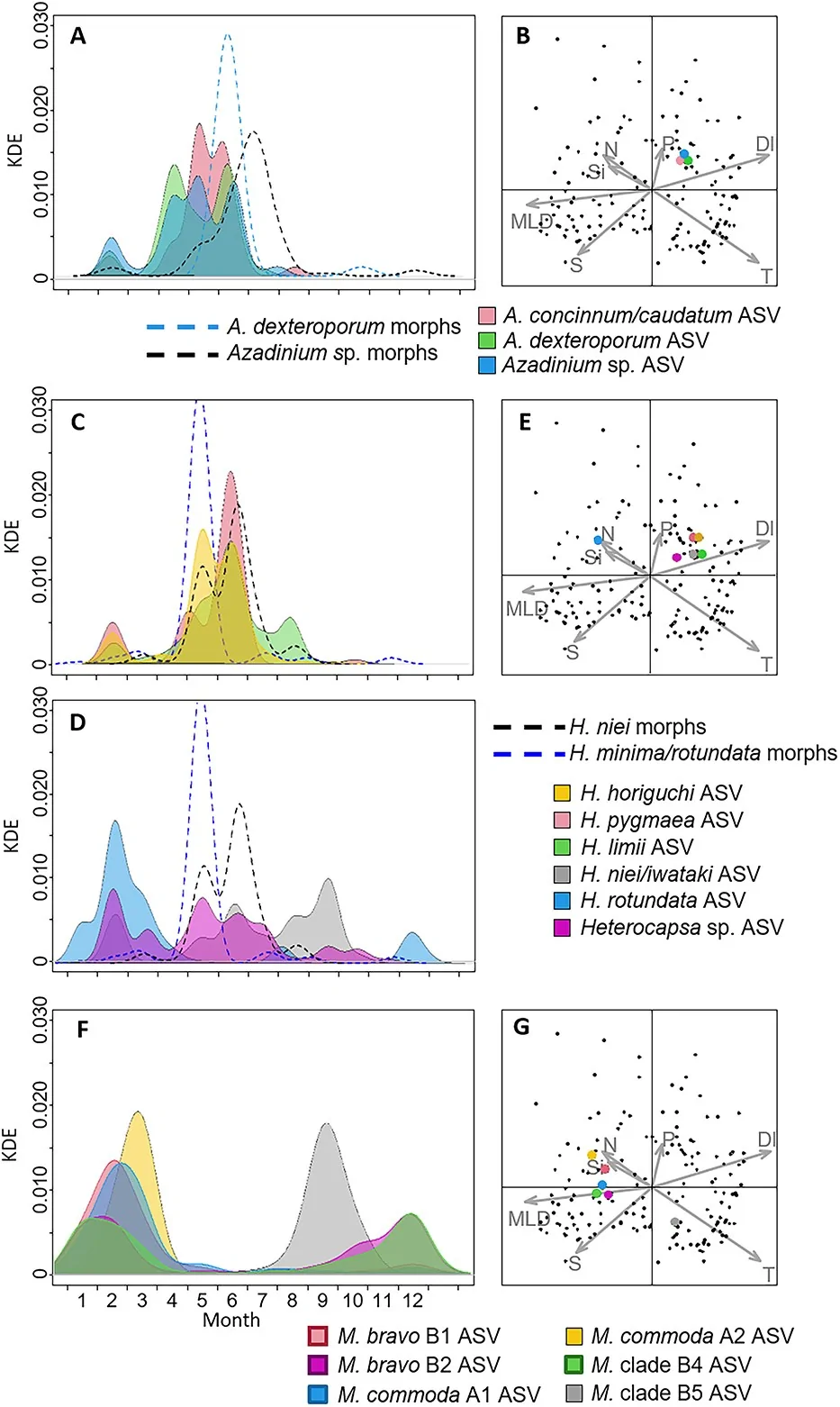
Seasonality, as KDE curves, and ecological niche of the *Azadinium* (A, B), *Heterocapsa* (C, D, E), and *Micromonas* (F, G) species complexes. Dashed lines represent the KDE curves of morphological species from LM data. Ecological niches (B, E, G) are represented on the common environmental space defined by the first and second axes of the OMI analysis (Fig. 2). Niche positions are represented by coloured dots (see legend for colour code), with larger distances of the dots from the plane centre indicating more specialized niches. Arrows represent the environmental variables characterizing the ecological niche. T, temperature; S, salinity; MLD, mixed layer depth; N, nitrate concentration; P, phosphate concentration; Si, silicate concentration; Dl, daylength. See Fig. 2 for the canonical weights of the environmental variables and Table S1 for OMI parameters and niche statistical significance.

The seasonal and ecological contexts are depicted in the OMI analysis, which identified two main gradients in the ‘common environmental space’ occupied by the targeted taxa. The first OMI axis (71.5% of the variation) counterposed December–January, characterized by low temperature, near-complete mixing of the water column, and higher salinity, to June–July, with long daylength, shallow mixed layer, and warmer temperatures. The second OMI axis captured 23% of the variation and counterposed winter–spring, with waters richer in nutrients, to summer–autumn, characterized by higher temperature and salinity and nutrient-poor waters (Fig. 2). With a few exceptions, taxa within each complex occupied distinct positions in the common environmental space (Figs 3–5, Table S1). Differences in the seasonal distribution and ecological preferences of the ASVs within the species complexes were also shown by the IQRs of their total abundance and of the corresponding environmental characteristics (Table S2). The niche parameters of the 33 taxa considered in the OMI analysis (Table S1) and the IQR values of the physical and chemical variables associated with their occurrence (Table S2), respectively, showed a high percentage residual tolerance and wide ranges of environmental conditions under which most taxa thrived.

The KDE, a smooth probability estimate of the population density along temporal and environmental gradients, allowed us to compare the distributions of both ASV and morphs of different abundances at the same scale (Figs 3–5) and calculate their seasonal and ecological niche overlaps (Table S3). The SO of taxa pairs ranged between 0.01 and 0.93 across all the species-complexes (0.43 ± 0.033, mean ± standard error), being lower than 0.6 (60% overlap) in 72% of the couples of sister taxa, and higher than 0.8 (80% overlap) only in 5 couples. The ecological NOs, calculated as an average of the KDE overlaps for the different environmental variables (Fig. 1), ranged between 0.28 and 0.89 (0.58 ± 0.020). The NO mean was largely influenced by the niche overlap for nutrients and salinity, the two variables generally not showing large differences across the seasons (Table S3).

**Figure 6:**
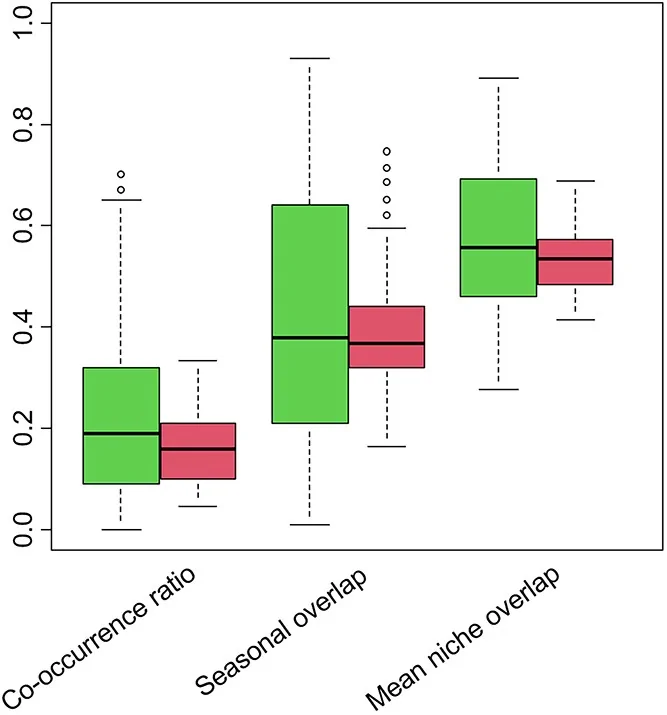
Co-occurrence ratio, seasonal overlap, and niche overlap values of cryptic and pseudocryptic species and genetic variant pairs (left boxes, in green) compared with values obtained from randomly selected taxa pairs (right boxes, in red). None of the indexes calculated for cryptic taxa was significantly different (*P* > .60) from those calculated for taxa pairs chosen at random.

PCA showed a strong correlation between SO and NO (Fig. S3), but NO was significantly higher than SO (*P* < .001; Fig. 6). High seasonal overlaps (SO >0.8) obviously coincided with high niche overlaps (NO >0.75) in the same taxa pair, while the couples with low SO (<0.20) did not have similarly low NO (range 0.28–0.51, average 0.39, Table S3). This is not totally unexpected for a temperate site with moderate environmental variations across the seasons. On the other hand, CoOCs of taxa pairs were much lower, ranging between 0 and 0.70 (0.24 ± 0.025), and were not related to SO and NO, but rather to ASV abundance and frequency, both affecting the probability of detecting taxa (Fig. S3, Table  S3).

None of the indices calculated for taxa pairs within species complexes was significantly different (*P* > .60) from those calculated on taxa pairs chosen at random (Fig. 6), which had CO 0.15 ± 0.017, SO 0.39 ± 0.009, and NO 0.53 ± 0.009.

The match between taxa records in the LM and molecular datasets (Co-OBservation rate, CoOB), based on presence-absence data, was generally low (0.24 ± 0.0345), because the high detection power of metabarcoding allowed the recording of rare taxa that were often undetected in LM analyses. CoOB was 0 or low for rare (e.g. *P. pseudodelicatissima*) or hardly identifiable taxa (e.g. *Azadinium*), and high (≤0.93) only in the case of very frequent or abundant species, e.g. *Leptocylindrus aporus*. Within species complexes, seasonal overlaps between morph and ASV abundances calculated from KDE curves (SoLM 0.11–0.82) attained values of ca. 0.6 or more for one or a few most abundant ASVs accounting for the largest part of the blooms recorded in LM (Table S4, Figs 3–5, Fig. S5). The other less abundant ASVs often showed different seasonalities, and hence scarce overlap with the morphs observed in LM. The rarefaction depth of the molecular dataset did not affect these results, as SoLM and CoOB showed little change between rarefaction depths of 30 000, 60 000, and 100 000 reads for most species, but higher differences at a depth of 10 000 reads (Fig. S4). A rarefaction depth of 30 000 reads seems a satisfying compromise between the accuracy of the calculated parameters and the number of samples (165 out of 168) included in the rarefaction.

In the following sections, we present each species complex describing the seasonality and niche characteristics of the distinct ASVs (Figs 3–5, Table S2), along with their co-occurrence, seasonal overlap, and niche overlap (Tables S3). Morphological and metabarcoding data are also compared to explore the correspondence between distribution patterns of cryptic taxa and morphs (Fig. S5, Table  S4).

#### Chaetoceros curvisetus/pseudocurvisetus complex

The four ASVs of this complex only co-occurred at times (mean CoOC 0.29), and diverged in both seasonality (mean SO 0.34) and ecological niche (mean NO 0.52, Fig. 3B), the less diverging couple being *C. curvisetus* 1 and *Chaetoceros pseudocurvisetus* (SO 0.52 and NO 0.68, Table S3). Minimum niche overlap (NO 0.38) occurred between *C. curvisetus* 2b and *C. curvisetus* 1, the former restricted to the extremely shallow MLD period (90% of its reads present in MLD < 11.9 m, Table S2) and matching the summer peak and the latter the winter peak of *C. curvisetus*-morphs (Fig. 3A, Fig. S5A). A third lineage, *C. curvisetus* 3, was scarcely abundant and found mainly in autumn. The *C. pseudocurvisetus* ASV was detected every year in autumn, and, in recent years, also in early spring, different from the *C. pseudocurvisetus*-morphs which were missed in some years (Fig. S5A).

#### Leptocylindrus

Four ASVs matching *Leptocylindrus* reference sequences were analysed, including one erroneously annotated as *Leptocylindrus danicus* str23 but in fact identical to a *Leptocylindrus hargravesii* reference and manually renamed (Table 1, Fig. S2B). Seasonality and niche characteristics varied among species of this complex. *L. aporus* ASV, the most abundant (75% of the genus reads) and frequent (93% of the samples), was found in summer and spring, matching the main and secondary peaks of *L.* cf. *danicus*-morphs (Fig. 3C, Table S4, Fig.  S5B). *L. danicus* ASV showed two annual peaks, in spring and autumn. *L. hargravesii* ASV was rarer (<7.1% of samples), with summer maximum but also present in December–January (relative abundance up to 25%), rarely co-occurring with the sister species *L. danicus* (SO 0.22; CoOC 0.10). *L. convexus* ASVs matched the *L. convexus-*morph pattern in summer. (CoOB 0.57, SoLM 0.82). The average ecological niche overlap of the four ASVs was 0.56, the highest match of 0.68 occurring between *L. aporus* and *L. hargravesii*, which both peaked in warmer, saltier, and nutrient-poorer conditions (Fig. 3D, Table S2). *L. danicus* occupied quite a large niche as environmental conditions markedly differed between spring–summer and autumn peaks (Fig. S6).

#### Pseudo-nitzschia delicatissima complex

Three ASVs assigned to the *P. delicatissima* complex were analysed (Table 1): two matched *P. delicatissima* and *P. allochrona*, respectively, while one assigned to *Pseudo-nitzschia dolorosa* and other species absent from the GoN was renamed as *Pseudo-nitzschia arenysensis/dolorosa* (Table 1). An abundant ASV matching *P. dolorosa* at 99.74% was pooled with *P. allochrona* because it is an intraspecific variant [31]. ASVs in this complex formed two distinct units that matched the first and second period of growth of the *P. delicatissima*-morphs, respectively. The first was formed by *P. arenysensis/dolorosa* and *P. delicatissima* ASVs, which frequently overlapped in March–April (SO 0.78, CoOC 0.63; Fig. 4A) and had similar ecological niches (NO 0.80). The other was *P. allochrona* ASV found in summer–autumn, never co-occurring with *P. arenysensis/dolorosa* and rarely with *P. delicatissima* (CoOC 0.02), and occupying a different niche (NO 0.43 and 0.51 with *P. arenysensis/dolorosa* and *P. delicatissima*, respectively), in warm, nutrient-depleted, and highly stratified waters (Fig. 4B; Table S2).

#### Pseudo-nitzschia galaxiae

Three frequent and abundant ASVs annotated as *P. galaxiae* followed one another throughout the year, occurring in February–March (*P. galaxiae* B571), March–May (B606), and July–September (B617). The former two partially matched the distribution of *P. galaxiae* small morphs (CoOB 0.43 with B571 and SoLM 0.69 with B606), while the latter corresponded to most of the medium and large morphs observed in summer (SoLM 0.62, Fig. 4C). *P. galaxiae* B571, rarely co-occurring with the others (CoOC 0.06 and 0.09), occupied a distinct niche from *P. galaxiae* B617 (NO 0.28), while *P. galaxiae* B606 showed no preferences for any environmental conditions (Fig. 4D, Table S1).

#### Pseudo-nitzschia pseudodelicatissima complex

In this complex, four frequent and abundant ASVs matched at 99.5%–100% the reference sequences of *P. pseudodelicatissima*, *Pseudo-nitzschia calliantha, Pseudo-nitzschia mannii*, and *P. caciantha/linea*, the latter two manually reassigned (Table 1). The couples *P. caciantha/linea-P. pseudodelicatissima* and *P. mannii*-*P. calliantha* had similar seasonalities (SO 0.73 and 0.87, respectively, Fig. 4E), but rarely co-occurred (CoOC 0.07 and 0.10, respectively), mostly due to the lack of records of one or the other in some years (Fig. S5E). The seasonality of the *P. caciantha/linea* ASV was more similar to that of *P. linea*-morphs (SO 0.60) than to that of *P. pseudodelicatissima*-morphs (0.48). Both *P. caciantha/linea* and *P. pseudodelicatissima* ASVs throve in late-winter nutrient-rich conditions (Table S2, Fig. 4F), while *P. mannii* and *P. calliantha* occupied nearly identical niches (NO 0.87) in warmer and more stratified waters, NO mean between species of the two couples being 0.48.

#### Azadinium

Of the three *Azadinium* ASVs analysed (Table 1), ASV 1386 matched *Azadinium dexteroporum,* ASV 540 matched both *Azadinium concinnum* and *Azadinium caudatum*, while ASV 560, attributed to *Azadinium* sp., matched the sequences of at least five different species (Fig. S2D). The assignments could not be refined manually because *Azadinium* were rarely and irregularly reported in LM observations (Fig. S5F). The three *Azadinium* ASVs peaked in April–June (SO 0.70–0.82, Fig. 5A) and shared a similar ecological niche (NO 0.71–0.81, Fig. 5B), although *A. concinnum/caudatum* records were too few to describe its niche (Table S1).

#### Heterocapsa

Six out of 36 ASVs attributed to *Heterocapsa* were analysed (Table 1), and their annotation was refined through phylogenetic analysis (Fig. S2E). *Heterocapsa limii*, *Heterocapsa horiguchii*, and *Heterocapsa pygmaea* presented a peak in May–June (Fig. 5C-D, mean SO 0.77 for the three), with largely overlapping ecological niches in stratified and warm waters (NO 0.67–0.77), and coincided with the *Heterocapsa niei*-morphs (SoLM 0.71–0.79, Table S3). By contrast, *Heterocapsa rotundata* peaked in February–March (mean SO and CoOC with other ASVs 0.24 and 0.04, respectively) and occupied a different ecological niche (mean NO with the other ASVs 0.40) in cold, mixed, and nutrient-richer waters (Fig. 5E, Table S2). Two other lineages, *H. niei/iwatakii* and *Heterocapsa* sp., lacked a clear seasonality. The latter was closely related to *H. rotundata* (Fig. S2E) and was abundant and frequent from March to June, and in some years until September (Fig. S5G).

#### Micromonas

Of 27 ASVs attributed to *Micromonas*, six belonging to distinct species or variants were studied (Table 1). Two ASVs attributed to *Micromonas pusilla* were renamed *Micromonas commoda* A2 and *Micromonas bravo* B2 [53], while no *M. pusilla* was recorded (Fig. S2F). With the exception of clade B5, present in a very restricted period of September, all lineages peaked in winter (Figs 5F, S5H, Table S2), congruent with *M. pusilla*-morphs studied in LM by serial dilution cultures [63]. Clade B4 and *M. bravo* B2 were both present in autumn–winter (SO 0.93) and had highly similar ecological niches (NO 0.89) but did not always co-occur (CoOC 0.65). *M. bravo* B1, the most abundant, and *M. commoda* A1 occurred both in February–March (SO 0.90, NO 0.82, Fig. 5F). *M. commoda* A2 had a more restricted seasonality than *M. commoda* A1 (SO 0.70), being present mostly in a short period of March (Table S2), and their co-occurrence was only 0.32. While Clade B5 had a distinct ecological niche in longer days and warm and stratified waters, the other four *Micromonas* variants broadly overlapped (NO 0.46–0.89, mean 0.67, Fig. 5G).

## Discussion

In spite of the low resolution attributed to 18S rDNA-V4, many sibling phytoplankton species were detected in our long-term metabarcoding dataset, thus providing insights into their diversity and distribution. Distinction is possible between phylogenetically close species pairs, such as *L. danicus*-*L. hargravesii* and *P. delicatissima*-*P. allochrona*, and even among genetic variants, e.g. within *C. curvisetus* and *P. galaxiae*. Cryptic species and variants can also be traced in small or fragile taxa not recorded in routine observations, such as *Micromonas* spp. and small featureless dinoflagellates. Clearly, our analysis does not cover the whole diversity of the target complexes. In fact, some ASVs slightly different from named reference sequences might be unknown or reference-lacking species, particularly in poorly known dinoflagellate genera, and would deserve in-depth investigation [4, 5]. Further, the 18S rDNA V4 fragment is not always effective in discriminating populations or cryptic species, which can be detected with other markers, as in the case of distinct *L*. *aporus* lineages identified by 18S rDNA V9 metabarcoding in the GoN [64]. The V4 fragment at times is even shared by species of the same genus, including non-cryptic ones (e.g. in the genera *Pseudo-nitzschia*, *Azadinium*, and *Heterocapsa*). In these cases, integrating knowledge of local diversity can refine the interpretation of metabarcoding results. For example, some ASVs shared by several species of the *P. delicatissima* complex are here confidently annotated as *P. arenysensis/dolorosa* because the alternative species, *P. arctica* and *P. subcurvata,* have a strictly polar distribution (Arctic and Antarctic, respectively) [31] and have never been found in the Gulf of Naples. This manual curation is obviously impossible without robust background knowledge which is missing, for example, in the case of small dinoflagellates.

Metabarcoding data are considered non-quantitative because of their compositional nature [65]. Yet, the annual distributions of the ASV abundance do show recurrent seasonal patterns across the years and, in each species complex, at least one abundant ASV matches relatively well the seasonal pattern of the corresponding morph. Therefore, normalized ASV abundances can be used effectively to describe the seasonality of many cryptic taxa, which would not be possible using only presence/absence data. In the case of taxa previously studied using a few years of molecular data [27, 29], long-term metabarcoding data confirm their seasonal patterns and corroborate the view that a regular annual recurrence is a widespread characteristic of phytoplankton species in the GoN [34]. Yet new and more detailed information emerges in some cases, e.g. for the phenology of *L. convexus* and *L. hargravesii*, having their annual peaks in summer rather than in winter [28, 32], and *P. delicatissima,* broadly co-occurring with *P. arenysensis* [31], but slightly later in the year. These new results highlight the power of long metabarcoding time-series, which integrate interannual variability and provide a more reliable description of species seasonality.

Seasonal patterns for some species of the GoN are similar to those observed elsewhere. *H. rotundata* is a winter species also in many temperate and subtropical estuaries [66–68]. The hypothesis that it prefers waters stratified by freshwater inputs [69] is not valid for the mixed winter water column of the GoN, whereas its ability to use phagotrophy at reduced irradiance levels might be more important [70]. Likewise, several *Micromonas* lineages occur in winter at other Atlantic [71] and Mediterranean sites [72]. A mismatch in seasonality is instead seen in *Micromonas* clade B5, observed in the Bay of Banyuls in late November [72], two months later than in the GoN, despite similar environmental conditions at the two sites. In such cases, different phenologies and niches support the idea of adaptations of cosmopolitan phytoplankton to local conditions, as postulated based on LM data [73]. Overall, the regular seasonal patterns of cryptic species can be exploited to complement LM species identification, but because of local adaptation processes this potential needs to be explored case by case in different areas.

Cryptic species should co-occur, or at least thrive under similar environmental conditions, based on the common assumption that both morphological similarity and phylogenetic relatedness correspond to similar physiological characteristics, ecological requirements, and functional roles [74, 75]. Contrary to this expectation, only 5 cryptic species pairs over 57 have highly similar ecological niches (NO ≥0.8: *P. arenysensis/dolorosa* and *P. delicatissima*; *P. calliantha* and *P. mannii*; *A. dexteroporum* and *Azadinium* sp.; *Micromonas bravo* B1 and *M. commoda* A1; and *M.* clade B4 and *M. bravo* B2), whereas niche overlap between all couples of sibling species is on average not different from randomly picked couples of phytoplankton taxa, and co-occurrence rate is even lower. Thus, phylogenetic and morphological similarities do not seem to imply conservation of the ecological niche. Based on molecular data, significant distributional differences have also been reported for distinct clades of the picoprasinophytes *Ostreococcus* and *Bathycoccus* [76]. These observations are in accordance with LM results that show similar, congeneric species occurring throughout the year under different environmental conditions [77]. Presumably, similar morphologies encompass a wide variety of physiological characteristics, including light and nutrients affinity [78], which can be most important in determining the ecological niche of planktonic protists.

The environmental conditions driving the seasonality and niches of individual taxa do not emerge clearly from our results. The high relative residual tolerance in the OMI analyses suggests that the environmental variables considered might not always be the primary determinants of their occurrence, while other factors, e.g. intrinsic or extrinsic biological factors, should be taken into account. No information is available on possible differences in grazing pressure or other interspecific relationships that could affect the occurrence of closely related species. Yet their distinct seasonality, diverging more than their ecological niche and recurring across the years, matches the seasonal rhythms observed in numerous cases for phytoplankton species, which are related to the photoperiod [34] and not affected by the marked interannual fluctuations of associated environmental variables [72, 79]. Sharing stable phenological traits within a species is relevant to the survival of ephemeral organisms, as encounter probability and sexual reproduction rates rise if all taxon members bloom at the same time [80]. The timing of sexual reproduction, not necessarily matching bloom timing, has rarely been investigated (e.g. in the diatom *Pseudo-nitzschia multistriata* [81]), but can now be assessed by tracing signal of sex in metatranscriptomic databases [82]. In nutrient-rich and rapidly fluctuating environments like the GoN, to be present at the same time might be more important for fitness than to be able to cope with suboptimal abiotic or biotic conditions.

It remains to be assessed whether the phenological and ecological divergence among cryptic species may be specific to the GoN, where environmental filtering could be less effective in selecting certain life forms in different periods of the year [34]. This peculiarity of the study area would limit the possibility to transpose the information gathered there to other places. Yet, a comparable seasonal shift in intrageneric/intraspecific ASV occurrence has been reported for diatom communities of Narragansett Bay [83], a quite different site dominated by wide salinity fluctuations. In all cases, the observed ecological differences among cryptic species could offer new angles to the interpretation of complex seasonal patterns. For example, the seasonality shift observed in the *C. curvisetus-pseudocurvisetus* complex in the Adriatic Sea [84], interpreted as an adaptation of the same taxon to changing environmental conditions, could rather be the result of a replacement among cryptic species or genetic variants with different phenologies.

Phenological differentiation among sibling taxa might be linked to speciation processes, which in phytoplankton remain unclear [85]. Temporal divergence could result from speciation in different environments (e.g. at different latitudes) followed by secondary contact. In this case, in the new location a species would occupy the season most similar to its native range, as seen in the polar-temperate lineage *C. curvisetus* 1 and the tropical-temperate *C. curvisetus* 2b, found in the GoN in winter and summer, respectively [17]. Allochronic speciation, i.e. sympatric speciation based on temporal segregation [86], could also apply to some cases, as proposed for the cryptic *P. allochrona* and *P. delicatissima*, found in winter–spring and summer–autumn, respectively. [29]. Allochronic speciation would require a genetically rooted seasonality [86] and relatively weak biotic and abiotic environmental constraints on the species’ growth rate, as might be the case in the GoN, and would lead to a change of the ecological niche in response to a phenology shift. Alternatively, the colonization of new niches (or times of year) would be the consequence of the plasticity or genetic diversity within individual taxa, which would ultimately segregate and diverge as separate cryptic species. Subsequent phenological changes could follow allochronic or allopatric speciation and lead to the temporal co-occurrence of cryptic species observed in some cases in this study (e.g. the *P. delicatissima*-*P. arenysensis* couple or several *Micromonas* variants), and also reported in insects [87]. Observation-based studies like ours are inadequate to distinguish the ecological and evolutionary mechanisms behind seasonality, which should be addressed by exploring the genetic basis of phenology [88] and the environmental clues used by the species to initiate their blooms.

Widespread ecological and phenological divergences between cryptic taxa underscore an underestimated functional diversity concealed behind morphological stasis. Such diversity should be explicitly incorporated into trait-based ecological frameworks and projections of geographic ranges into future scenarios, often extrapolated from limited experimental data on a small number of strains. The persistence of morphologically similar taxa across diverse environmental conditions likely reflects multiple adaptive mechanisms, including physiological plasticity, intraspecific genetic variation, and local adaptation processes. Emerging approaches in comparative metagenomics and transcriptomics offer promising avenues to elucidate metabolic differences between sister species. Long-term metabarcoding datasets, particularly when combined with the use of more sensitive taxonomic markers, will be a powerful tool in uncovering novel cryptic diversity and delineating species-specific ecological niches. This enhanced resolution will improve our understanding of microbial adaptive potential and increase the reliability of predictive models aimed at assessing biodiversity responses to climate change.

## Supplementary Material

Web_Material_ycag225

## Data Availability

The data used to support the conclusions of this paper are mostly presented in the supplementary materials and tables. The chemical and physical data are part of the dataset analyzed in [34] and available at https://doi.org/10.5281/zenodo.7924734. FASTA of the ASVs, LM data, and other data not shown in the supplementary material are available on request.
