# Supplementary material for: Niche divergence suggests ecophysiological differences between cryptic phytoplankton species
Source: ISME Commun. 2026 Aug 4;6(1):ycag225. doi: 10.1093/ismeco/ycag225 (PMC13590887; doi:10.1093/ismeco/ycag225)
Supplement: Supplementary file 1 [file web_material_ycag225.zip › Supplementary figures 1-6_ycag225.pdf]

Niche divergence suggests ecophysiological differences between cryptic phytoplankton species

Nicolas Romillac[1], Gianpaolo Zampicinini[1], Adriana Zingone[1c], Diana Sarno[1,2c]

[1]Department of Research Infrastructures for Marine Biological Resources, Stazione Zoologica Anton Dohrn, Villa Comunale, 80121 Naples, Italy
[2]NBFC, National Biodiversity Future Center, Piazza Marina 61, 90133 Palermo, Italy

SUPPLEMENTARY FIGURES

Figure S1 Map of the Gulf of Naples (Tyrrhenian Sea, Mediterranean Sea) with the location of the sampling site LTER MareChiara (LTER-MC).

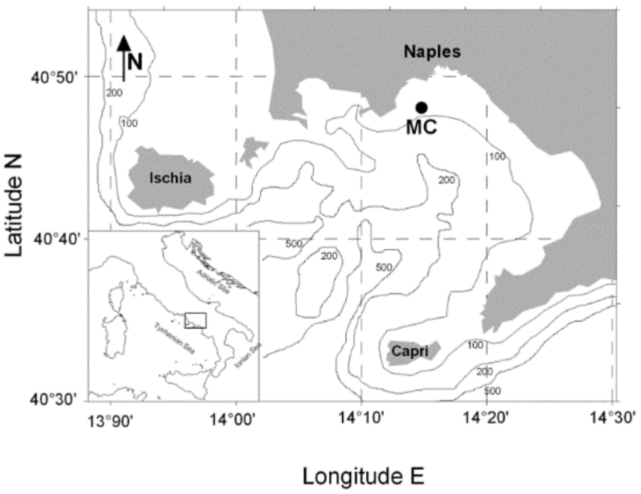

Figure S2 Phylogenetic trees (Maximum Likelihood, ML) of V4 sequences of target species (Table 1) and whole rDNA 18S reference sequences, identified by the GenBank accession number (identical to the first 10 characters of the PR2 accession number) and by species and strain name. ASVs retained in the ecological analyses are in bold; those matching different reference sequences are indicated by boxes of the same colour. Bootstrap values from 100 replicates are shown if >50. (A) *Chaetoceros* tree, with *Leptocylindrus danicus* as outgroup. (B) *Leptocylindrus* tree, with *Tenuicylindrus belgicus* as outgroup. (C) *Pseudo-nitzschia* tree, with *Cylindrotheca closterium* as outgroup. The name of strain NWSFC 185 was corrected according to Lundholm et al. (2012). (D) *Azadinium* tree, with *Heterocapsa pygmaea* as outgroup. (E) *Heterocapsa* tree, with *Scrippsiella trochoidea* (=*Scrippsiella acuminata*) as outgroup. Species names were corrected according to DinoRef (Mordret et al., 2018) and the taxonomic attribution in PR2, when different, is indicated between brackets. Sequences unambiguously attributed to *H. niei* and *H. triquetra* were also included. (F) *Micromonas* tree, with *Mamiella gilva* as outgroup. A sequence unambiguously attributed to *M. pusilla* was also included. Species names were changed according to Tragin and Vaulot (2019).

Figure S2 (A)

A

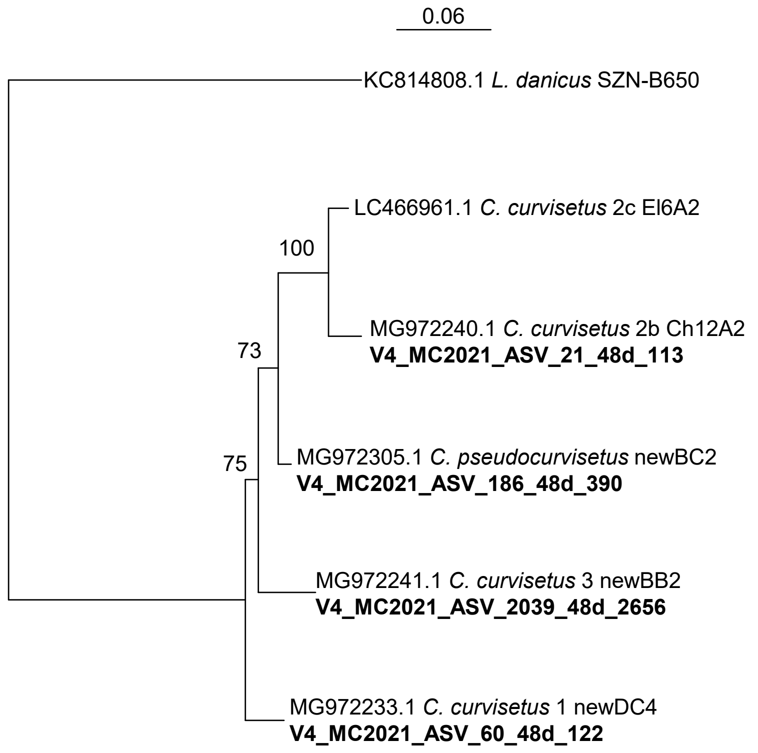

Figure S2 (B)

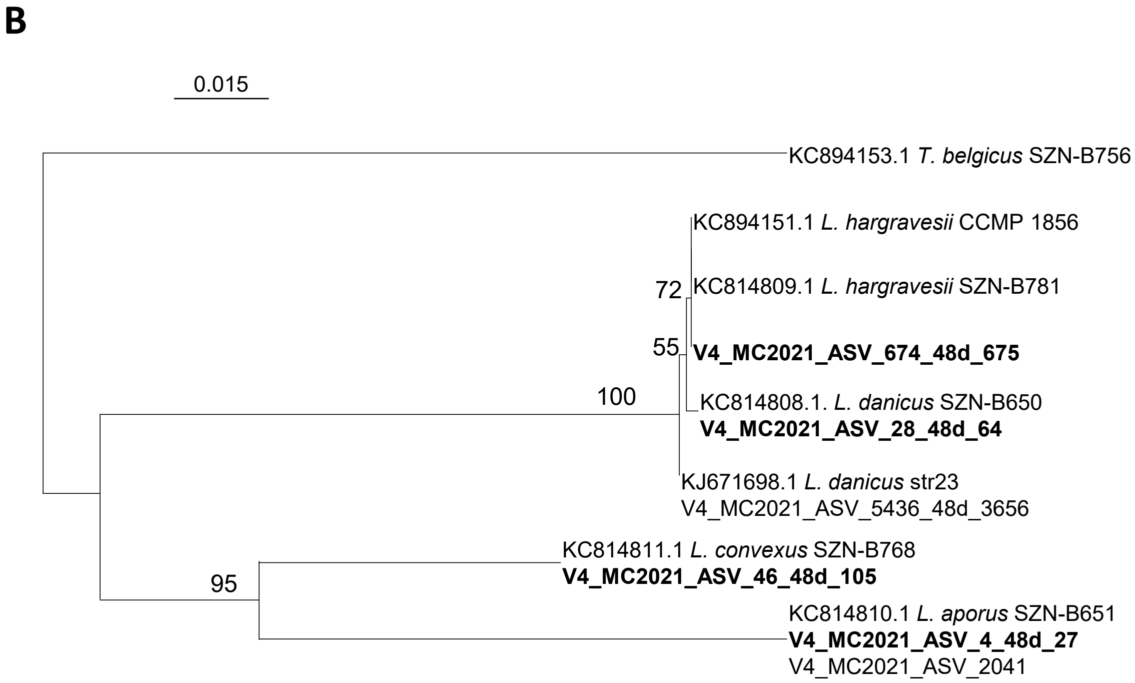
B
0.015
KC894153.1 T. belgicus SZN-B756
KC894151.1 L. hargravesii CCMP 1856
72
KC814809.1 L. hargravesii SZN-B781
55
V4_MC2021_ASV_674_48d_675
100
KC814808.1. L. danicus SZN-B650
V4_MC2021_ASV_28_48d_64
KJ671698.1 L. danicus str23
V4_MC2021_ASV_5436_48d_3656
KC814811.1 L. convexus SZN-B768
95
V4_MC2021_ASV_46_48d_105
KC814810.1 L. aporus SZN-B651
V4_MC2021_ASV_4_48d_27
V4_MC2021_ASV_2041

Figure S2 (C)

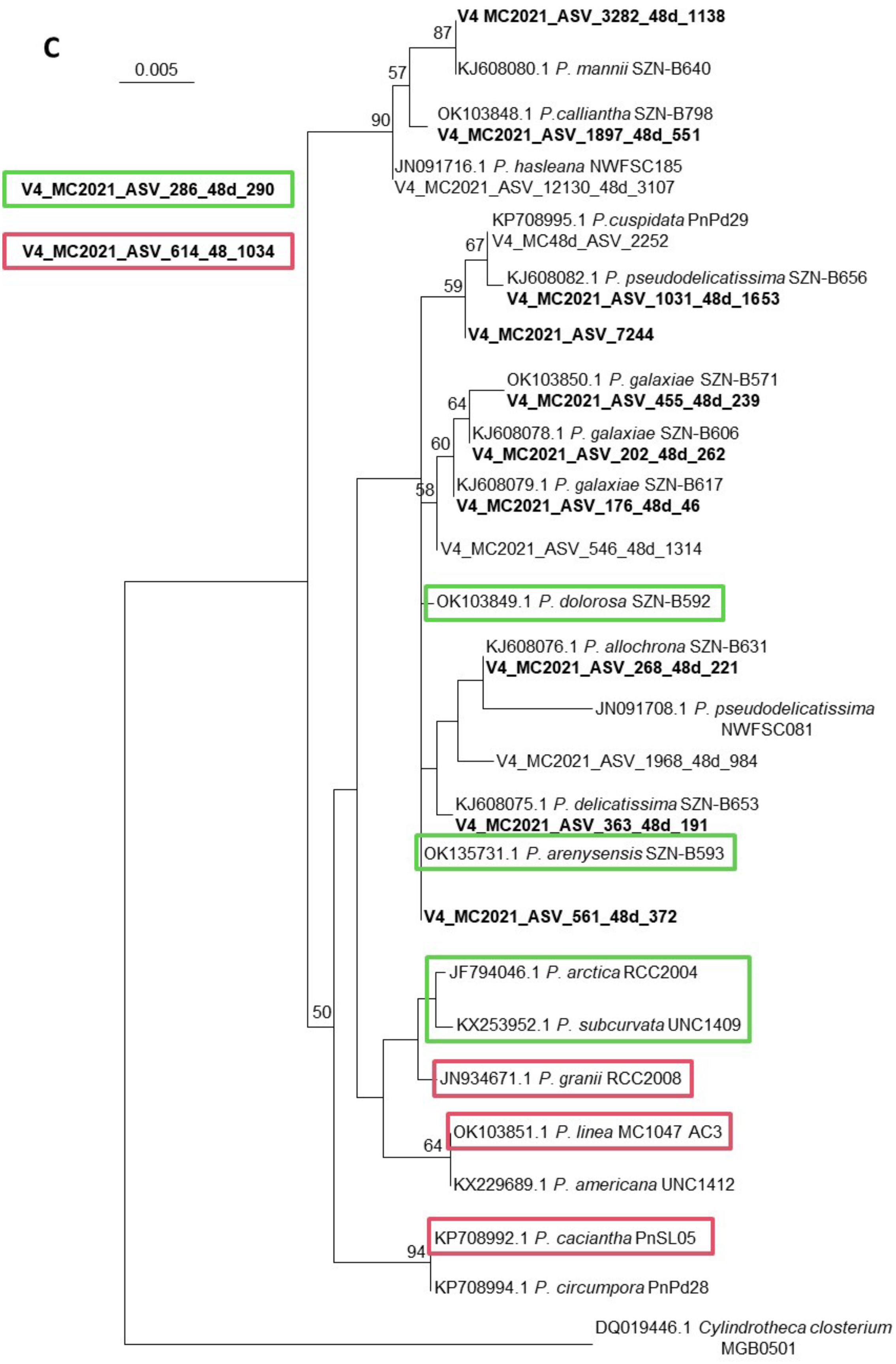

C
0.005
V4 MC2021_ASV_3282_48d_1138
87
KJ608080.1 P. mannii SZN-B640
57
OK103848.1 P.calliantha SZN-B798
90
V4_MC2021_ASV_1897_48d_551
JN091716.1 P. hasleana NWFSC185
V4_MC2021_ASV_12130_48d_3107
V4_MC2021_ASV_286_48d_290
V4_MC2021_ASV_614_48_1034
KP708995.1 P.cuspidata PnPd29
67
V4_MC48d_ASV_2252
59
KJ608082.1 P. pseudodelicatissima SZN-B656
V4_MC2021_ASV_1031_48d_1653
V4_MC2021_ASV_7244
OK103850.1 P. galaxiae SZN-B571
64
V4_MC2021_ASV_455_48d_239
KJ608078.1 P. galaxiae SZN-B606
60
V4_MC2021_ASV_202_48d_262
KJ608079.1 P. galaxiae SZN-B617
58
V4_MC2021_ASV_176_48d_46
V4_MC2021_ASV_546_48d_1314
OK103849.1 P. dolorosa SZN-B592
KJ608076.1 P. allochrona SZN-B631
V4_MC2021_ASV_268_48d_221
JN091708.1 P. pseudodelicatissima NWFSC081
V4_MC2021_ASV_1968_48d_984
KJ608075.1 P. delicatissima SZN-B653
V4_MC2021_ASV_363_48d_191
OK135731.1 P. arenysensis SZN-B593
V4_MC2021_ASV_561_48d_372
JF794046.1 P. arctica RCC2004
50
KX253952.1 P. subcurvata UNC1409
JN934671.1 P. granii RCC2008
OK103851.1 P. linea MC1047 AC3
64
KX229689.1 P. americana UNC1412
KP708992.1 P. caciantha PnSL05
94
KP708994.1 P. circumpora PnPd28
DQ019446.1 Cylindrotheca closterium MGB0501

Figure S2 (D)

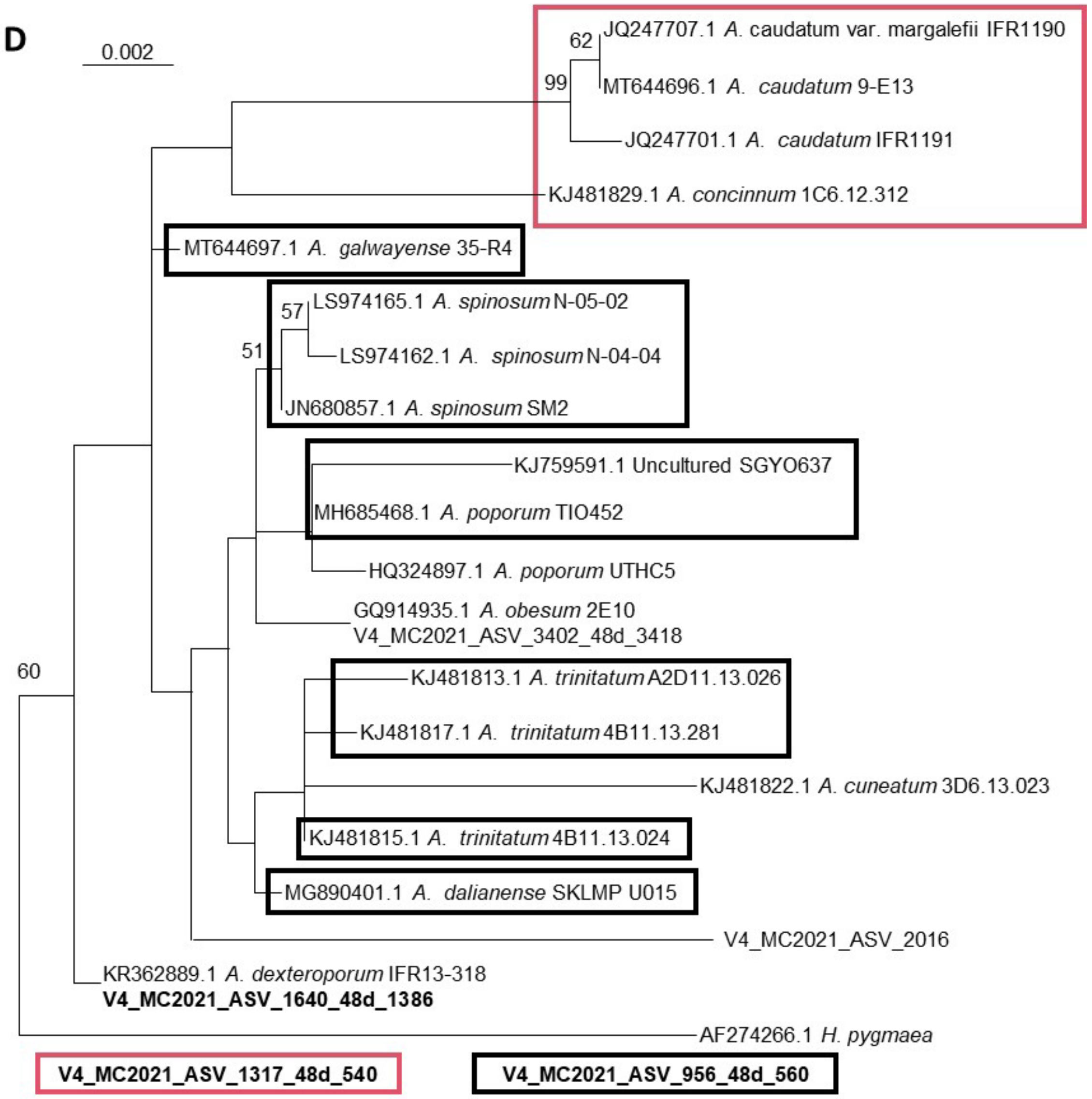
D
0.002
62
99
JQ247707.1 A. caudatum var. margalefii IFR1190
MT644696.1 A. caudatum 9-E13
JQ247701.1 A. caudatum IFR1191
KJ481829.1 A. concinnum 1C6.12.312
MT644697.1 A. galwayense 35-R4
57
51
LS974165.1 A. spinosum N-05-02
LS974162.1 A. spinosum N-04-04
JN680857.1 A. spinosum SM2
KJ759591.1 Uncultured SGYO637
MH685468.1 A. poporum TIO452
HQ324897.1 A. poporum UTHC5
GQ914935.1 A. obesum 2E10
V4_MC2021_ASV_3402_48d_3418
60
KJ481813.1 A. trinitatum A2D11.13.026
KJ481817.1 A. trinitatum 4B11.13.281
KJ481822.1 A. cuneatum 3D6.13.023
KJ481815.1 A. trinitatum 4B11.13.024
MG890401.1 A. dalianense SKLMP U015
V4_MC2021_ASV_2016
KR362889.1 A. dexteroporum IFR13-318
V4_MC2021_ASV_1640_48d_1386
AF274266.1 H. pygmaea
V4_MC2021_ASV_1317_48d_540
V4_MC2021_ASV_956_48d_560

Figure S2 (E, F)

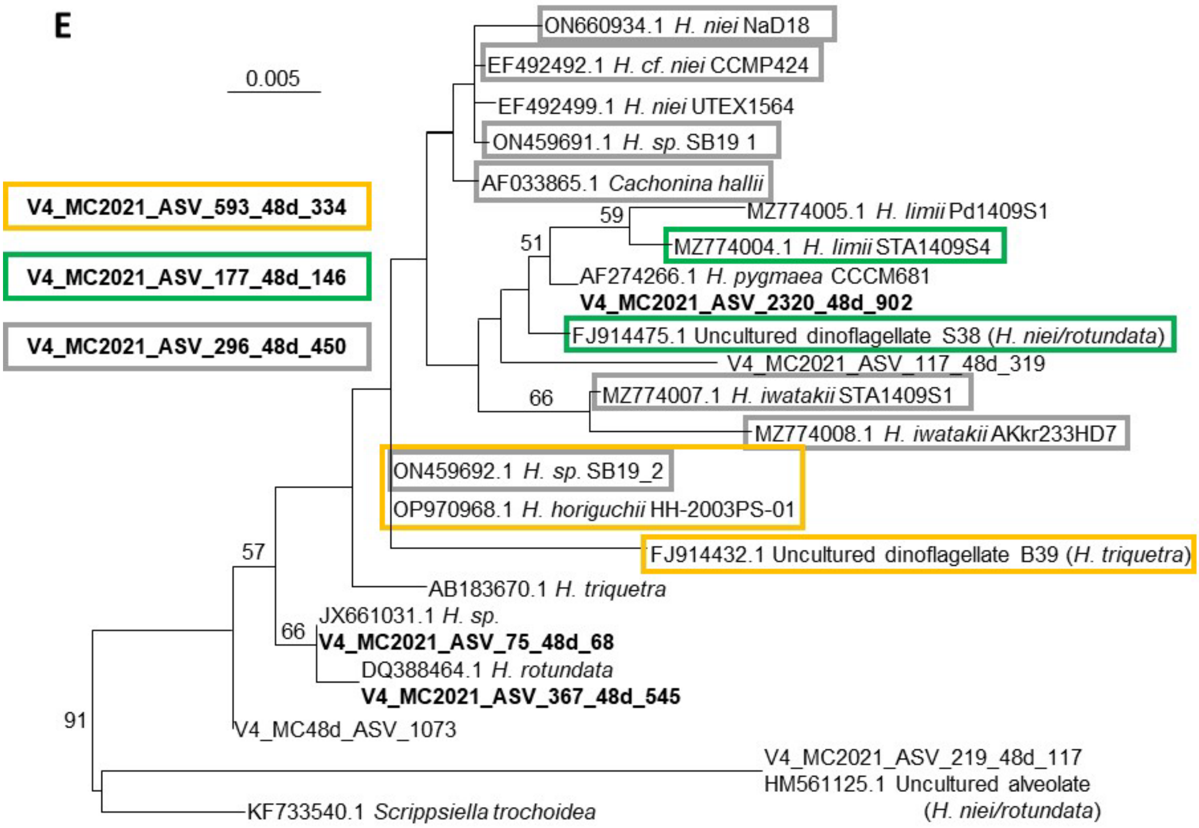
E
0.005
V4_MC2021_ASV_593_48d_334
V4_MC2021_ASV_177_48d_146
V4_MC2021_ASV_296_48d_450
ON660934.1 H. niei NaD18
EF492492.1 H. cf. niei CCMP424
EF492499.1 H. niei UTEX1564
ON459691.1 H. sp. SB19 1
AF033865.1 Cachonina hallii
59
MZ774005.1 H. limii Pd1409S1
51
MZ774004.1 H. limii STA1409S4
AF274266.1 H. pygmaea CCCM681
V4_MC2021_ASV_2320_48d_902
FJ914475.1 Uncultured dinoflagellate S38 (H. niei/rotundata)
V4_MC2021_ASV_117_48d_319
66
MZ774007.1 H. iwatakii STA1409S1
MZ774008.1 H. iwatakii AKkr233HD7
ON459692.1 H. sp. SB19_2
OP970968.1 H. horiguchii HH-2003PS-01
FJ914432.1 Uncultured dinoflagellate B39 (H. triquetra)
57
AB183670.1 H. triquetra
JX661031.1 H. sp.
66
V4_MC2021_ASV_75_48d_68
DQ388464.1 H. rotundata
V4_MC2021_ASV_367_48d_545
91
V4_MC48d_ASV_1073
V4_MC2021_ASV_219_48d_117
HM561125.1 Uncultured alveolate (H. niei/rotundata)
KF733540.1 Scrippsiella trochoidea

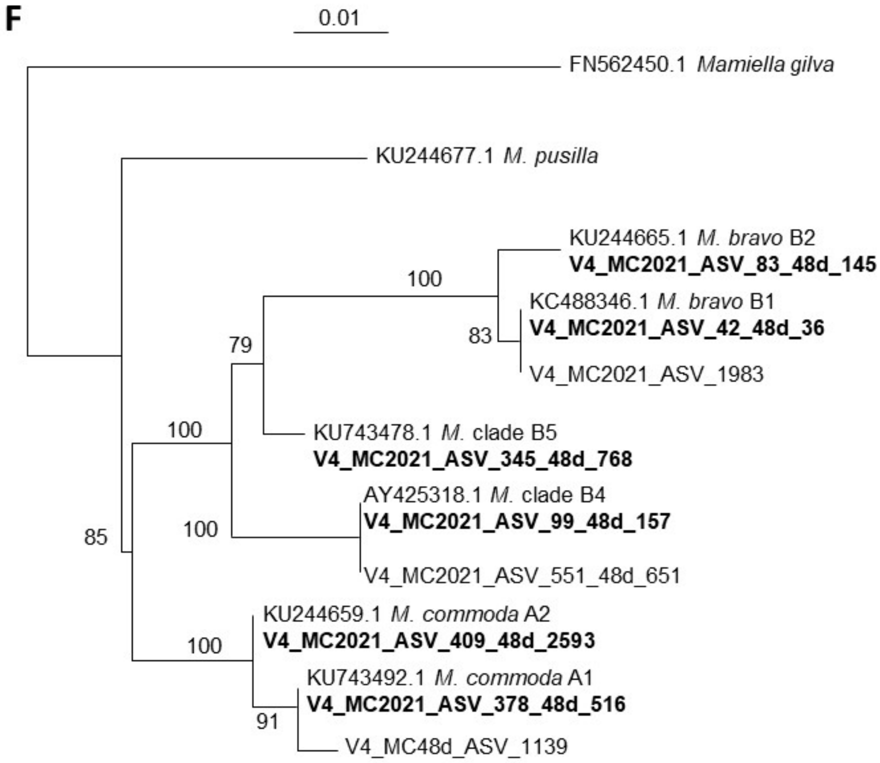
F
0.01
FN562450.1 Mamiella gilva
KU244677.1 M. pusilla
KU244665.1 M. bravo B2
V4_MC2021_ASV_83_48d_145
100
KC488346.1 M. bravo B1
V4_MC2021_ASV_42_48d_36
83
V4_MC2021_ASV_1983
79
KU743478.1 M. clade B5
V4_MC2021_ASV_345_48d_768
100
AY425318.1 M. clade B4
V4_MC2021_ASV_99_48d_157
100
85
V4_MC2021_ASV_551_48d_651
KU244659.1 M. commoda A2
V4_MC2021_ASV_409_48d_2593
100
KU743492.1 M. commoda A1
V4_MC2021_ASV_378_48d_516
91
V4_MC48d_ASV_1139

Figure S3 Principal Component Analysis (correlation biplot) of seasonal overlap, co-occurrence ratio and mean niche overlap of cryptic species pairs. The first two axes are presented. Abundance, as the sum of reads of species pairs, is used as a supplementary variable.

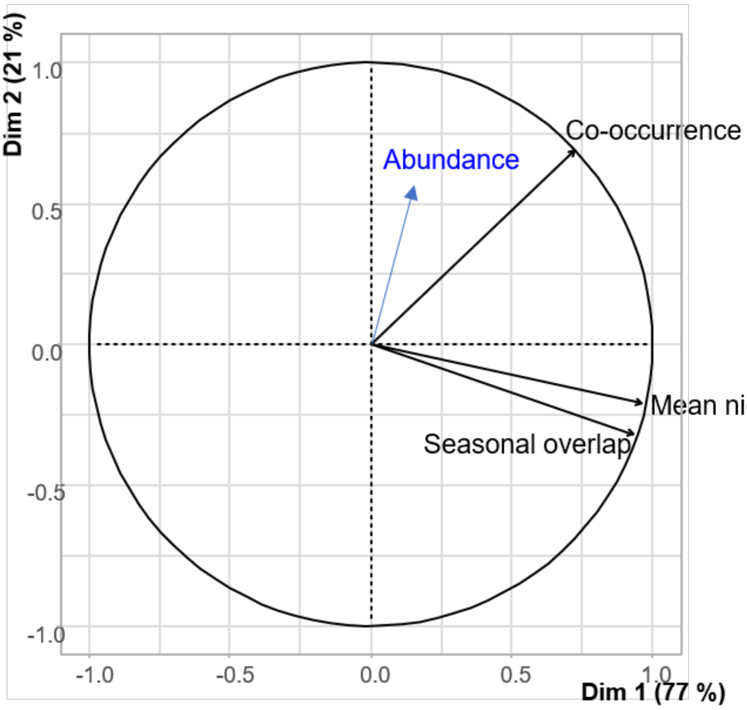

Figure S4 Seasonal overlap (SoLM) and co-observation rate (CoOB) between ASVs and corresponding morphs identified in Light Microscopy (LM) across different rarefaction depths. The sensitivity analysis was conducted on 88 sampling dates (out of 168) with > 100,000 reads. SoLM was calculated as the overlap between the KDE curves representing the seasonal distribution of each taxon in metabarcoding and LM datasets, respectively. CoOB is the ratio of samples in which one taxon was detected by both methods to samples in which it was detected by one or both methods. 0 indicates no co-observation, 1 co-observation on all sampling dates. Each line corresponds to one ASV-morph pair.

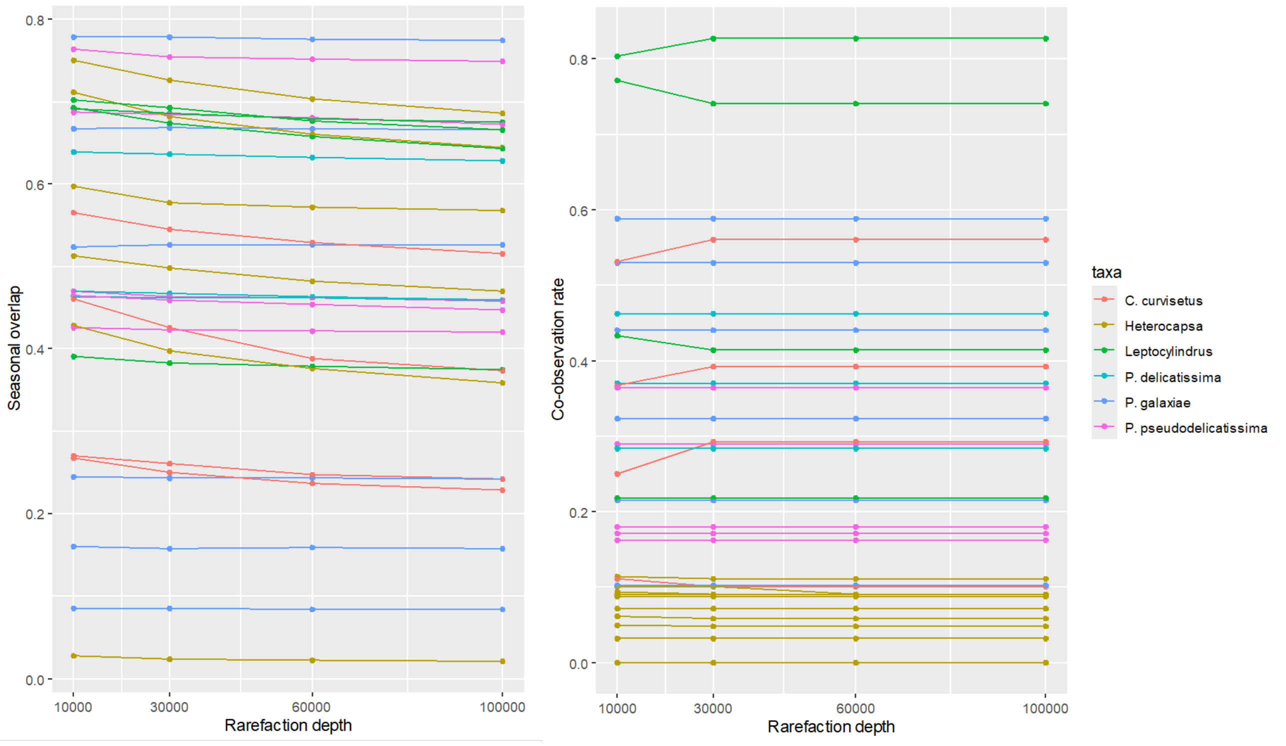

Figure S5 Heatmaps of ASV (metabarcoding, yellow to black color gradient) and morph (Light Microscopy,green to black color gradient) abundances per month and year, and ASVs' relative abundances per month. (A) *Chaetoceros curvisetus/pseudocurvisetus* complex. (B) *Leptocylindrus.* (C) *Pseudo-nitzschia delicatissima* complex. (D) *Pseudo-nitzschia galaxiae* complex. (E) *Pseudo-nitzschia pseudodelicatissima* complex. (F) *Azadinium.* (G) *Heterocapsa*. (H) *Micromonas*. ASV abundance is the number of reads in the dataset rarefied at 30,000 reads/sample. All values are monthly medians.

Figure S5

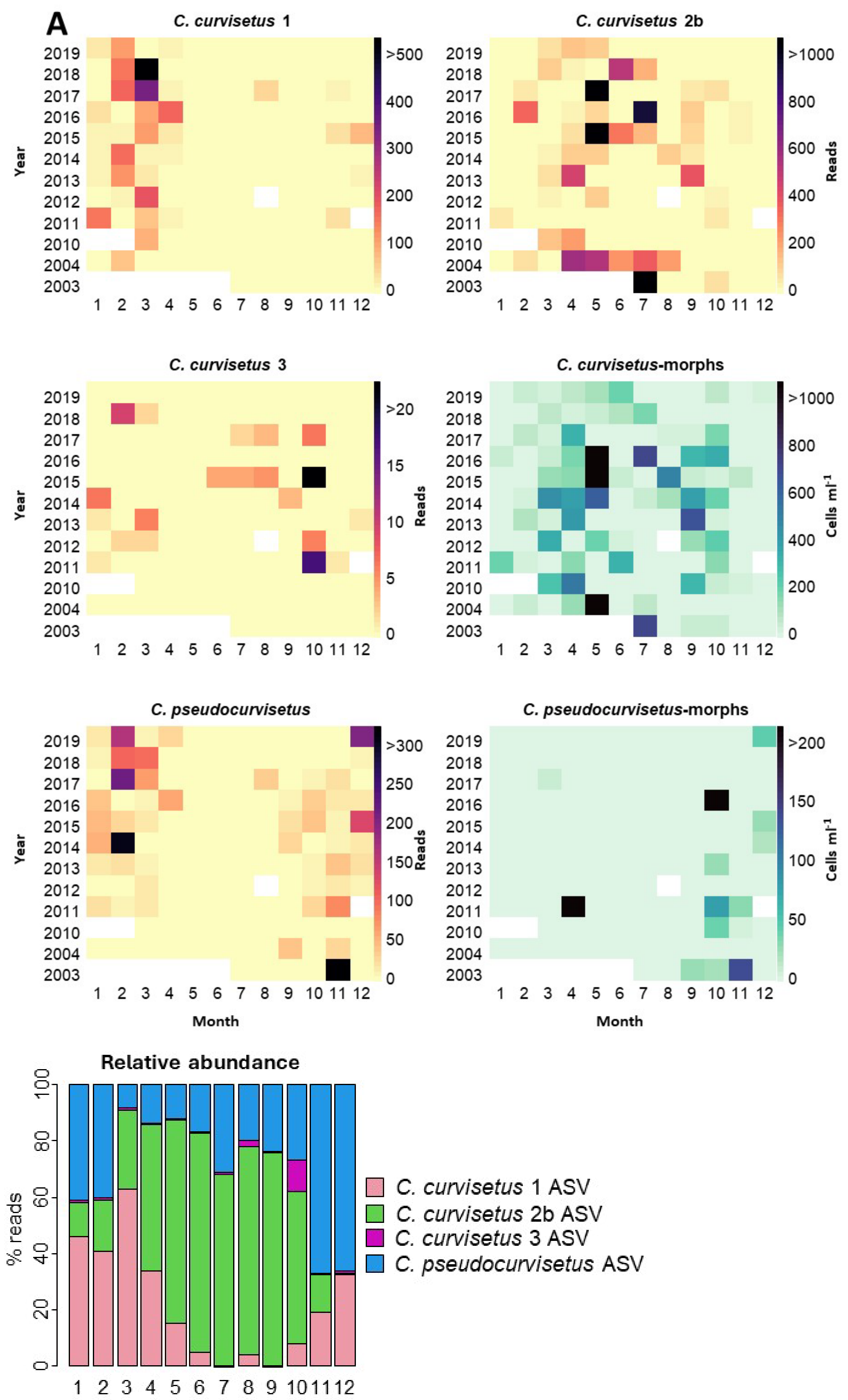

A
C. curvisetus 1
C. curvisetus 2b
C. curvisetus 3
C. curvisetus-morphs
C. pseudocurvisetus
C. pseudocurvisetus-morphs
Year
Reads
Cells ml-1
Month
2019
2018
2017
2016
2015
2014
2013
2012
2011
2010
2004
2003
1 2 3 4 5 6 7 8 9 10 11 12
>500
400
300
200
100
0
>1000
800
600
400
200
0
>20
15
10
5
0
>300
250
200
150
100
50
>200
150
100
50
Relative abundance
% reads
0
20
40
60
80
100
C. curvisetus 1 ASV
C. curvisetus 2b ASV
C. curvisetus 3 ASV
C. pseudocurvisetus ASV

Figure S5

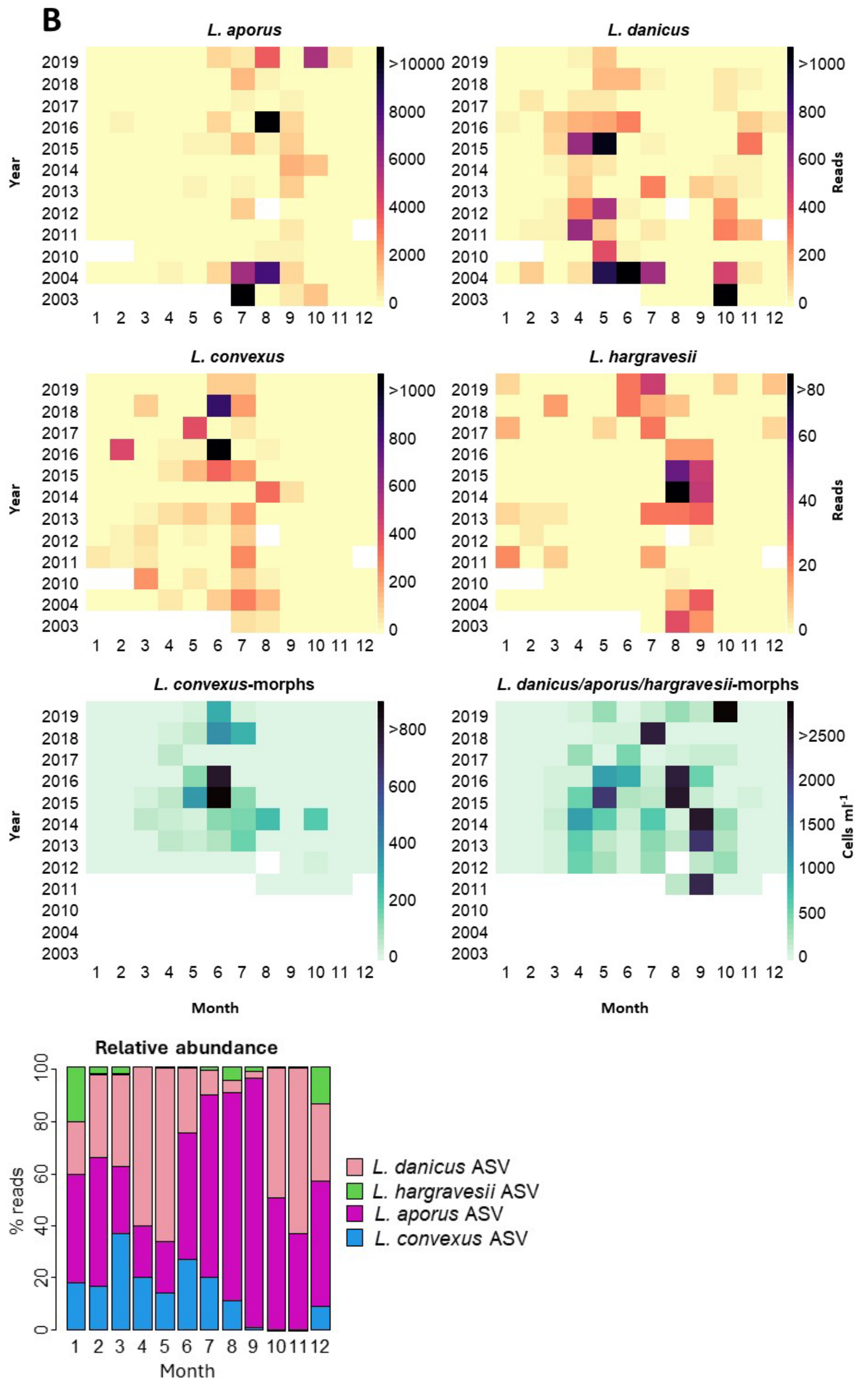
B
L. aporus
L. danicus
L. convexus
L. hargravesii
L. convexus-morphs
L. danicus/aporus/hargravesii-morphs
Year
Month
Reads
Cells ml-1
>10000
>1000
>80
>800
>2500
2019
2018
2017
2016
2015
2014
2013
2012
2011
2010
2004
2003
Relative abundance
% reads
L. danicus ASV
L. hargravesii ASV
L. aporus ASV
L. convexus ASV

Figure S5

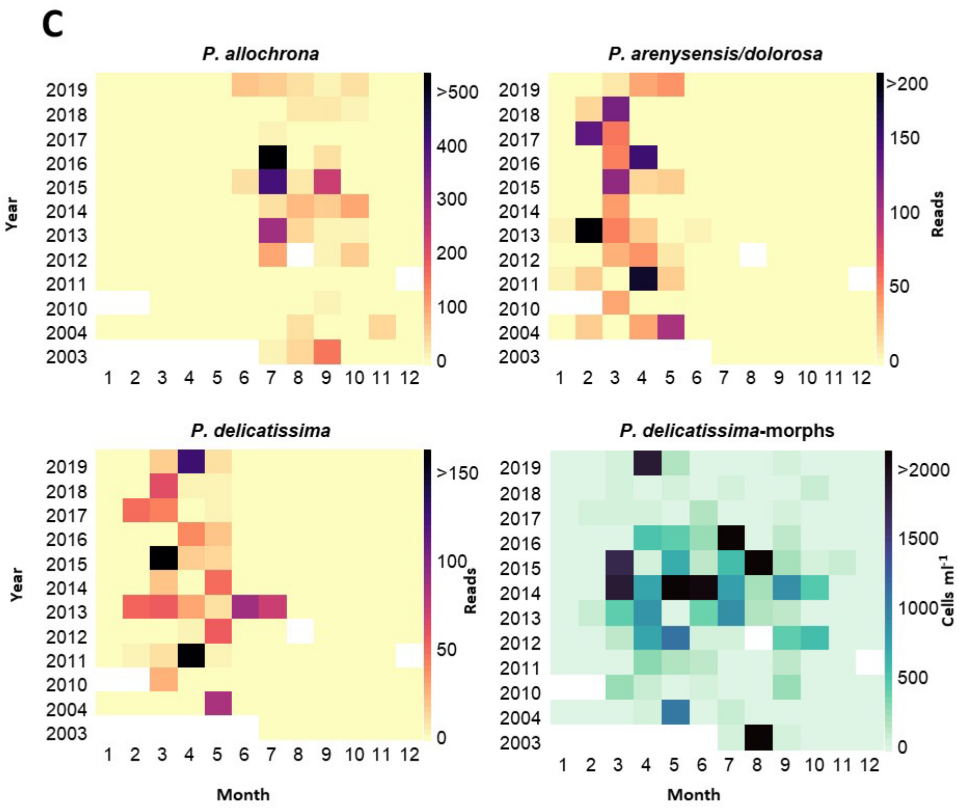
C
P. allochrona
P. arenysensis/dolorosa
P. delicatissima
P. delicatissima-morphs
Year
Month
Reads
Cells ml-1
>500
>200
>150
>2000

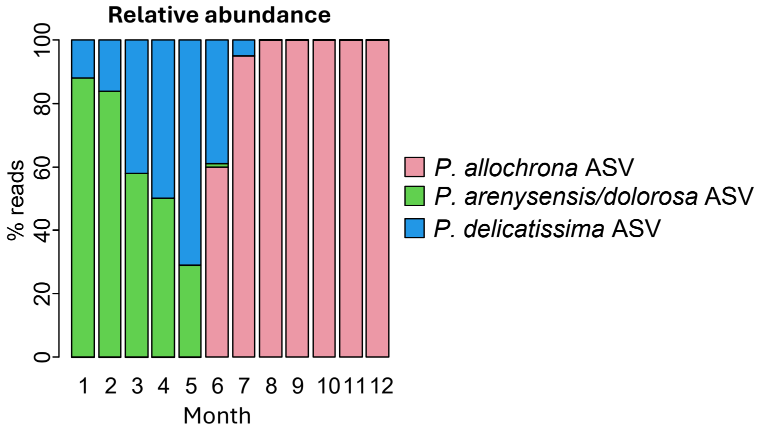
Relative abundance
% reads
Month
P. allochrona ASV
P. arenysensis/dolorosa ASV
P. delicatissima ASV

Figure S5

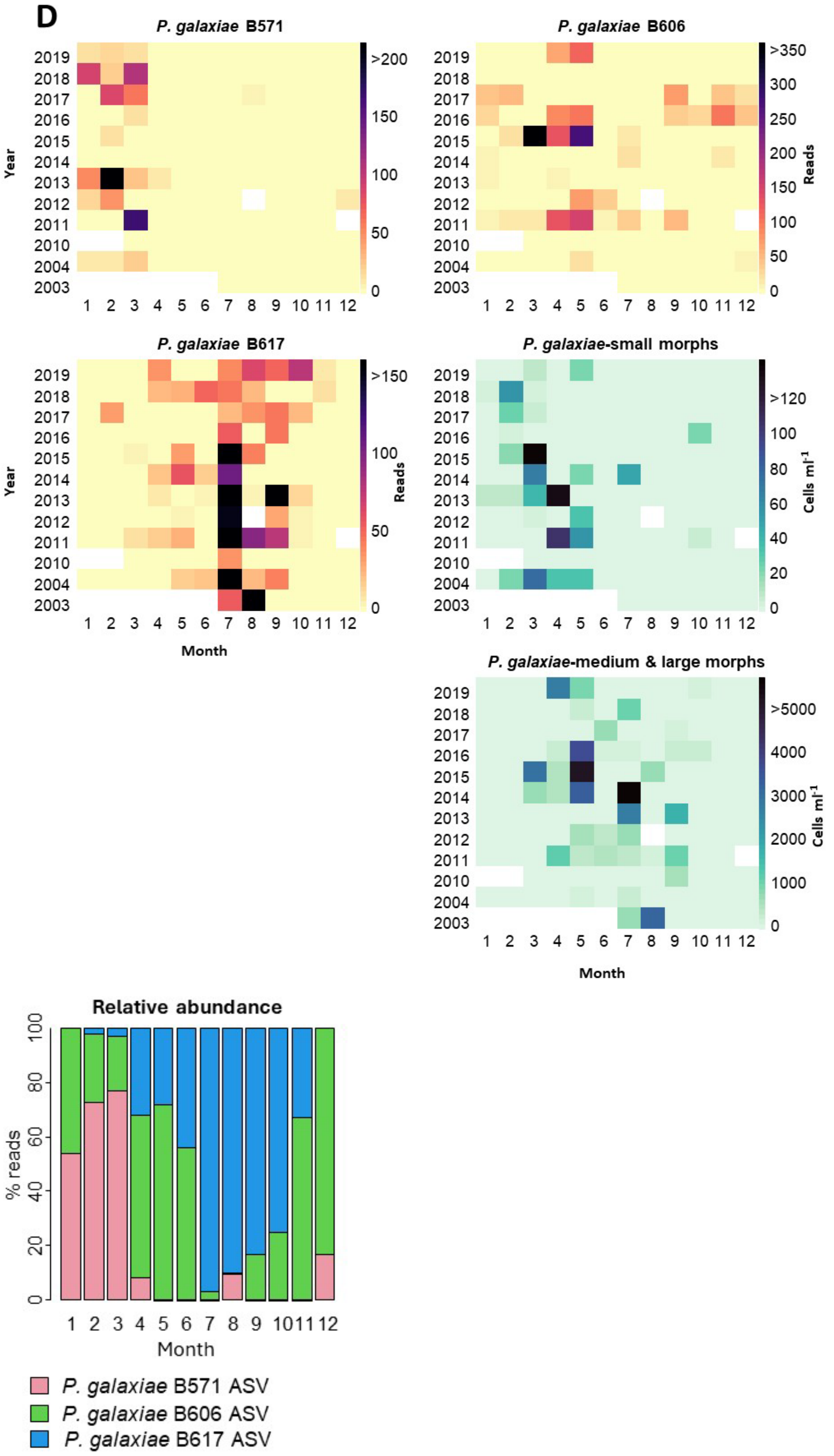
D
P. galaxiae B571
P. galaxiae B606
P. galaxiae B617
P. galaxiae-small morphs
P. galaxiae-medium & large morphs
Year
Month
Reads
Cells ml-1
2019
2018
2017
2016
2015
2014
2013
2012
2011
2010
2004
2003
1 2 3 4 5 6 7 8 9 10 11 12
>200
150
100
50
0
>350
300
250
200
>150
>120
80
60
40
20
>5000
4000
3000
2000
1000
Relative abundance
% reads
P. galaxiae B571 ASV
P. galaxiae B606 ASV
P. galaxiae B617 ASV

Figure S5

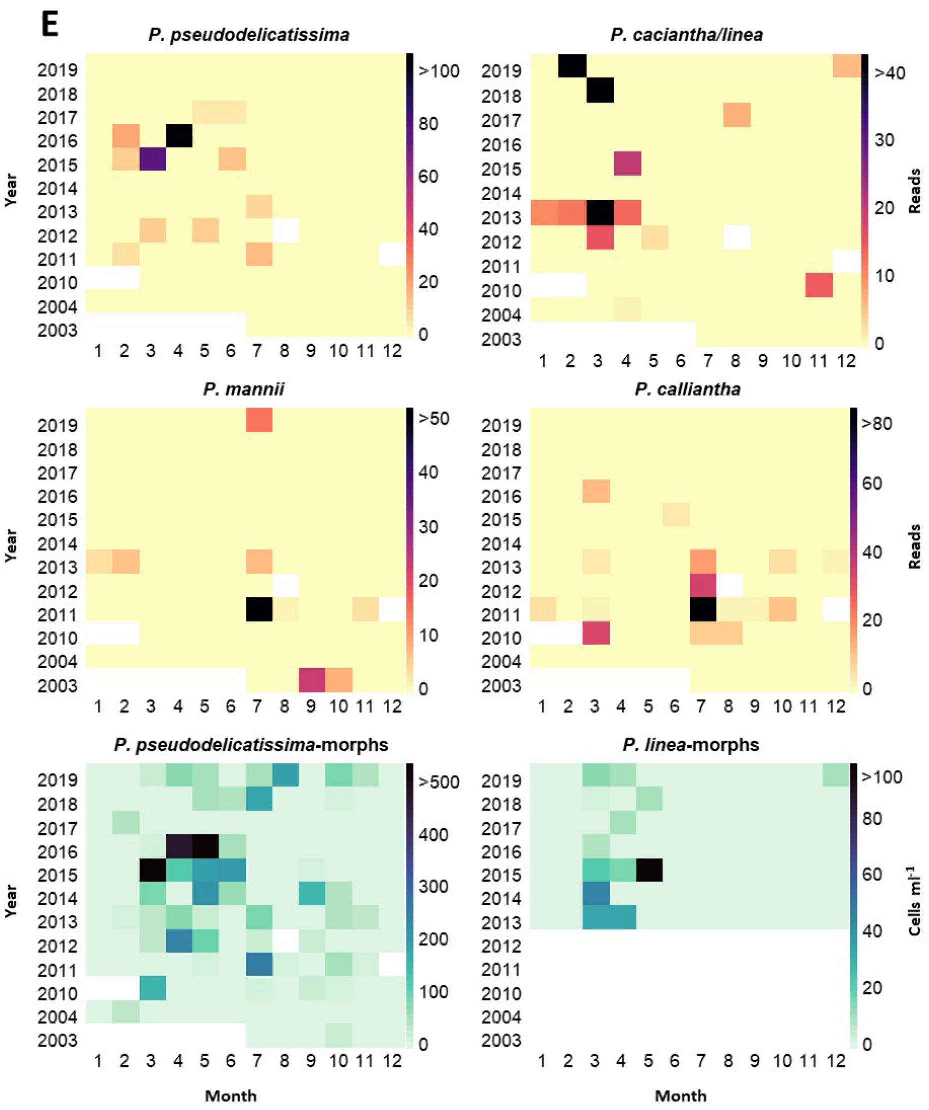
E
P. pseudodelicatissima
P. caciantha/linea
P. mannii
P. calliantha
P. pseudodelicatissima-morphs
P. linea-morphs
Year
Month
Reads
Cells ml-1

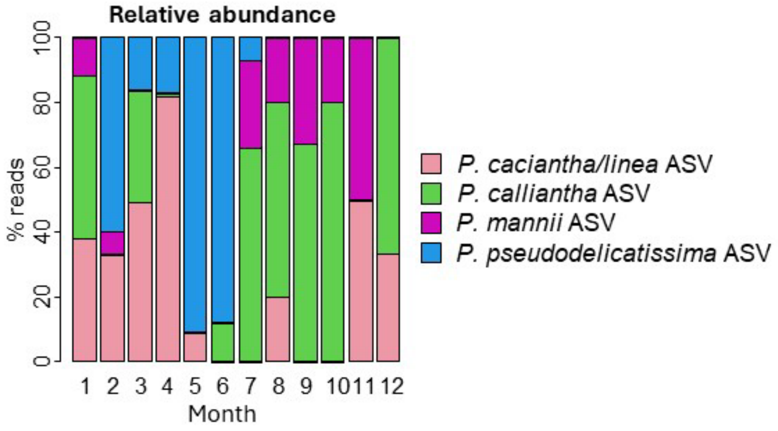
Relative abundance
% reads
Month
P. caciantha/linea ASV
P. calliantha ASV
P. mannii ASV
P. pseudodelicatissima ASV

Figure S5

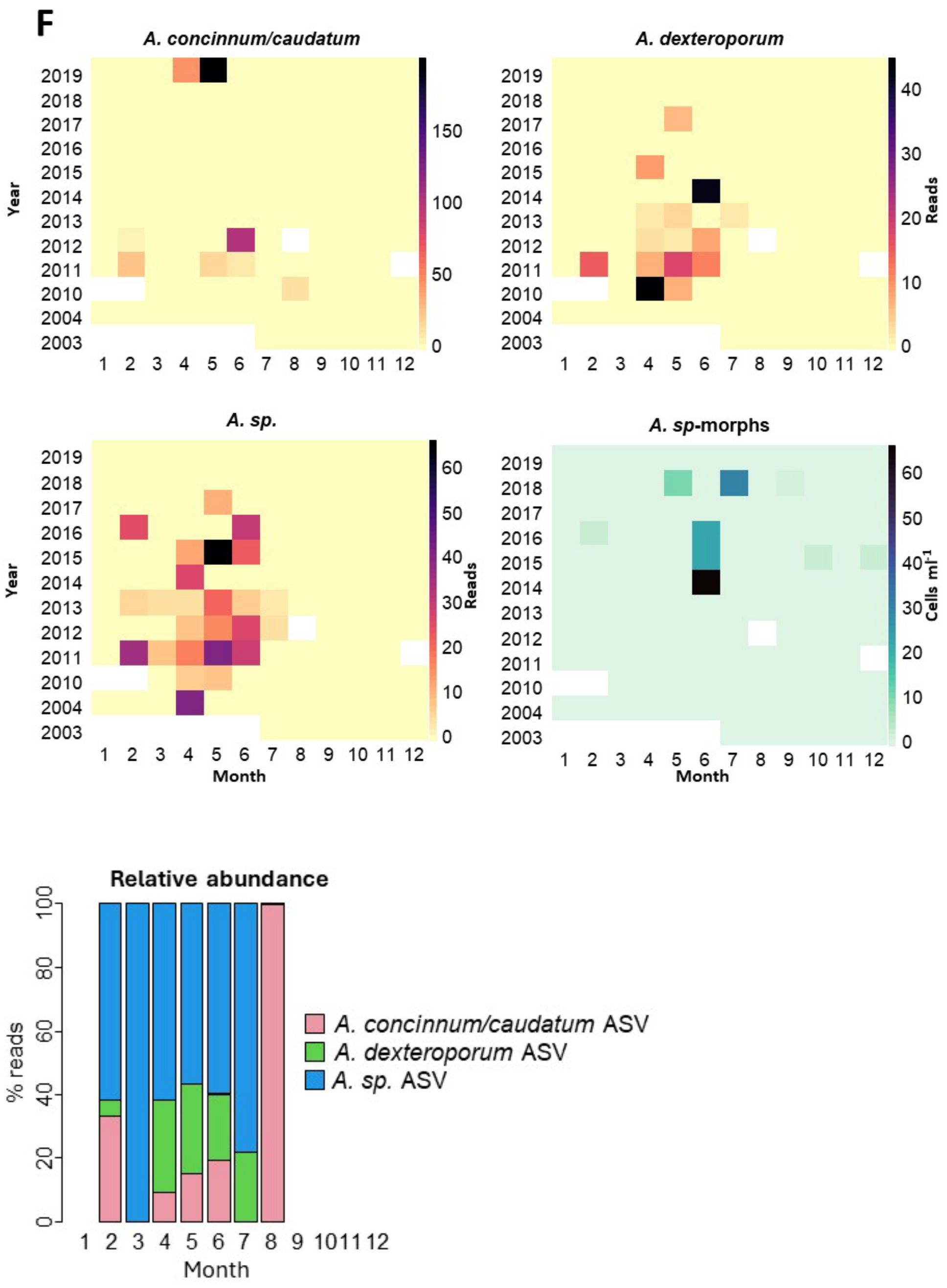
F
A. concinnum/caudatum
A. dexteroporum
A. sp.
A. sp-morphs
Year
Month
Reads
Cells ml-1
2019
2018
2017
2016
2015
2014
2013
2012
2011
2010
2004
2003
1 2 3 4 5 6 7 8 9 10 11 12
Relative abundance
% reads
A. concinnum/caudatum ASV
A. dexteroporum ASV
A. sp. ASV

Figure S5

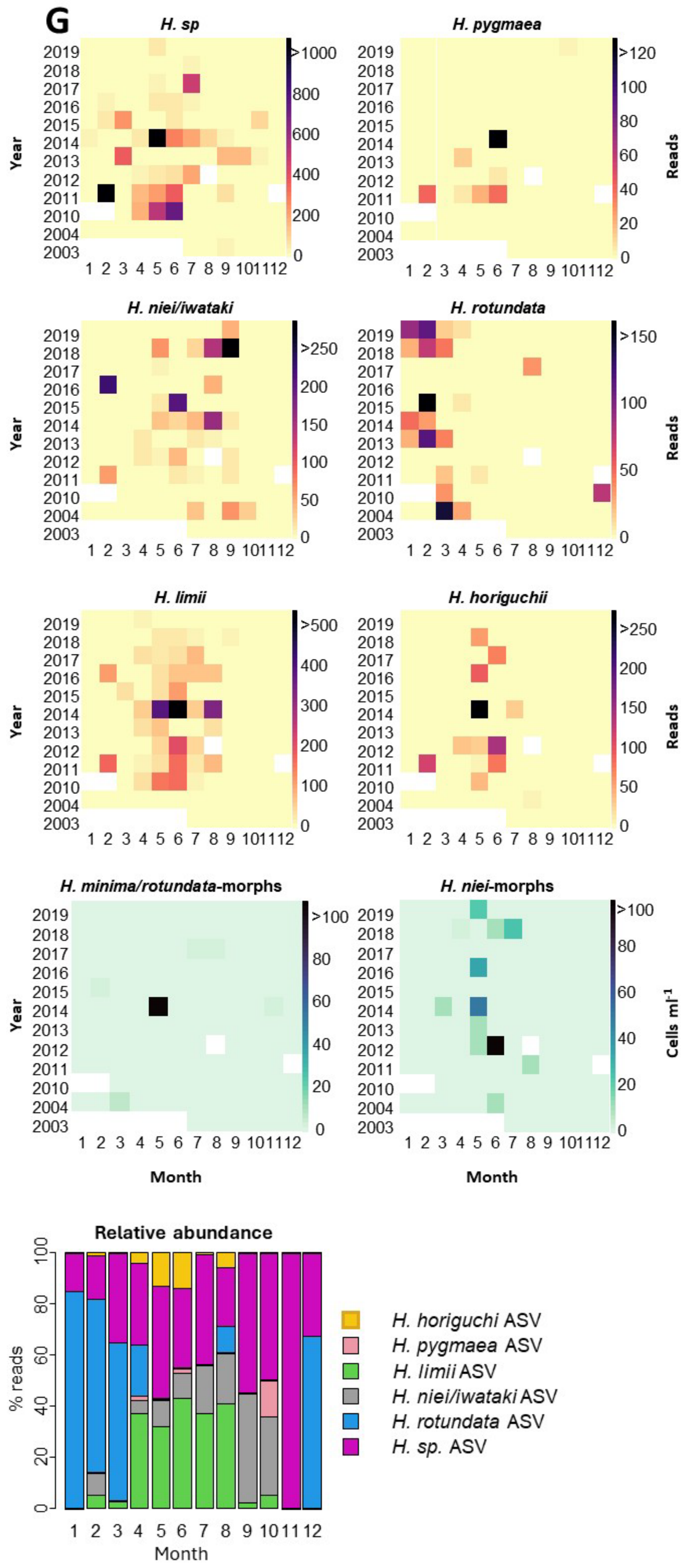

G
H. sp
H. pygmaea
H. niei/iwataki
H. rotundata
H. limii
H. horiguchii
H. minima/rotundata-morphs
H. niei-morphs
Year
Month
Reads
Cells ml-1
2019
2018
2017
2016
2015
2014
2013
2012
2011
2010
2004
2003
1 2 3 4 5 6 7 8 9 10 11 12
>1000
800
600
400
200
0
>120
100
80
60
40
20
>250
200
150
100
50
>150
>500
400
300
>100
Relative abundance
% reads
H. horiguchi ASV
H. pygmaea ASV
H. limii ASV
H. niei/iwataki ASV
H. rotundata ASV
H. sp. ASV

Figure S5

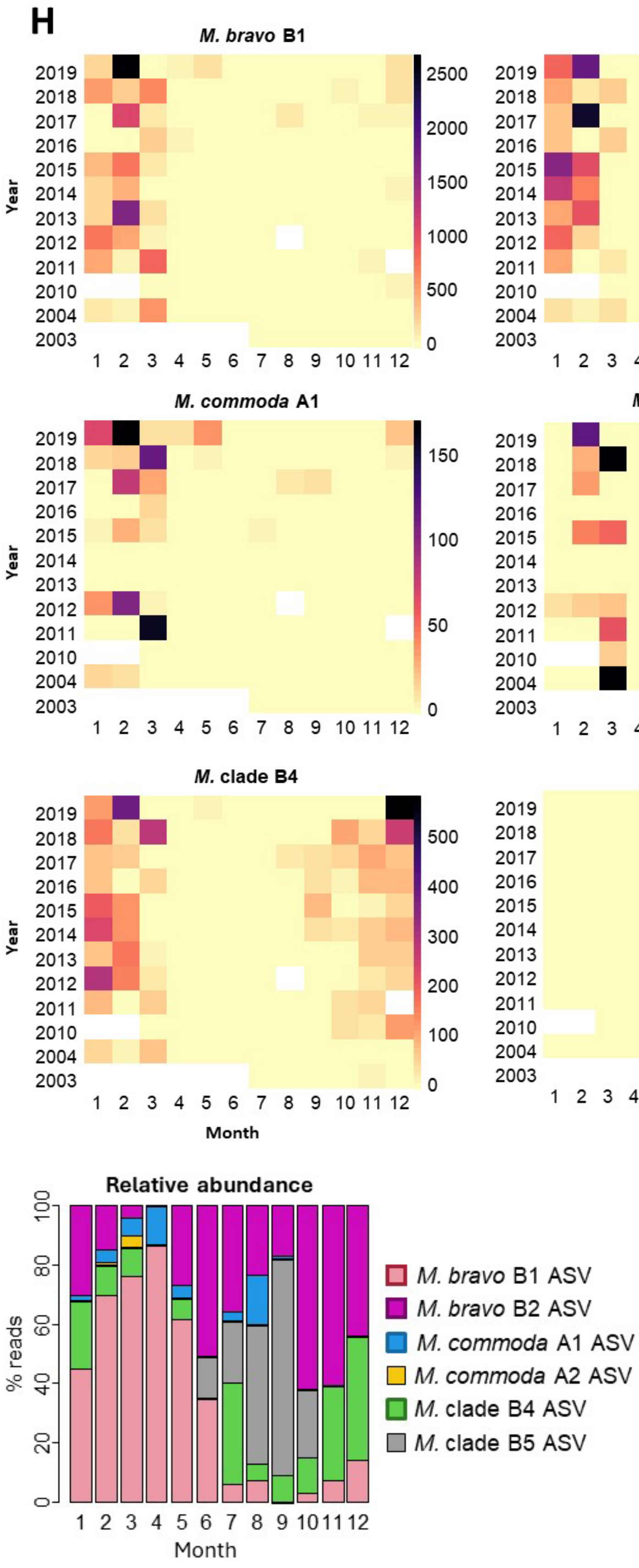
H
M. bravo B1
M. bravo B2
M. commoda A1
M. commoda A2
M. clade B4
M. clade B5
Year
Reads
Month
2019
2018
2017
2016
2015
2014
2013
2012
2011
2010
2004
2003
1 2 3 4 5 6 7 8 9 10 11 12
2500
2000
1500
1000
500
0
600
400
300
200
100
>50
40
30
20
10
>200
150
50
Relative abundance
% reads
M. bravo B1 ASV
M. bravo B2 ASV
M. commoda A1 ASV
M. commoda A2 ASV
M. clade B4 ASV
M. clade B5 ASV

Figure S6 Outlying Mean Index (OMI) analysis of the ecological niche of *Leptocylindrus danicus*, considering all seasons (red) and limited to spring-summer (February-July, blue) and autumn (September-December, green). T, temperature; S, salinity; MLD, mixed layer depth; N, nitrate concentration; P, phosphate concentration; Si, silicate concentration; Dl, Daylength. See Figure 1 for the canonical weights of the environmental variables.

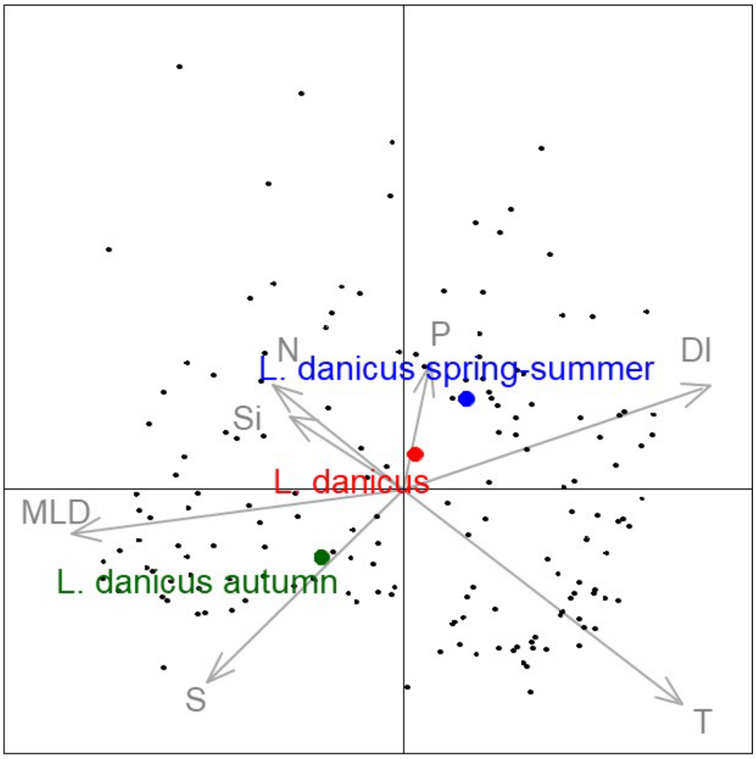

Literature

Lundholm N, Bates SS, Baugh KA, Bill BD, Connell LB, Léger C, & Trainer VL Cryptic and pseudo-cryptic diversity in diatoms—with descriptions of *Pseudo-nitzschia hasleana* sp. nov. and *P. fryxelliana* sp. nov. 1. *Journal of Phycology* 2012; ***48***: 436-454.

Mordret S, Piredda R, Vaulot D *et al.* DINOREF: A curated dinoflagellate (Dinophyceae) reference database for the 18S rRNA gene. *Mol Ecol Resour* 2018;**18**:974–87.

Tragin M, Vaulot D. Novel diversity within marine Mamiellophyceae (Chlorophyta) unveiled by metabarcoding. *Sci Rep* 2019;**9**, DOI: 10.1038/s41598-019-41680-6.
