# Supplementary material for: Niche divergence suggests ecophysiological differences between cryptic phytoplankton species
Source: ISME Commun. 2026 Aug 4;6(1):ycag225. doi: 10.1093/ismeco/ycag225 (PMC13590887; doi:10.1093/ismeco/ycag225)
Supplement: Supplementary file 1 [file web_material_ycag225.zip › Supplementary material Taxa description_ycag225.pdf]

Niche divergence suggests ecophysiological differences between cryptic phytoplankton species

Authors: Nicolas Romillac[1], Gianpaolo Zampicinini[1], Adriana Zingone[1c], Diana Sarno[1,2c]

[1]Department of Research Infrastructures for Marine Biological Resources, Stazione Zoologica Anton Dohrn, Villa Comunale, 80121

[2]NBFC, National Biodiversity Future Center, Piazza Marina 61, 90133 Palermo, Italy

[c]**Corresponding authors:**

Adriana Zingone: zingone@szn.it

Diana Sarno: diana.sarno@szn.it

Running head: niche divergence in cryptic phytoplankton species

SUPPLEMENTARY MATERIAL

Taxonomic and ecological notes on the species complexes and genera investigated

Chaetoceros curvisetus/*pseudocurvisetus* complex

Chaetoceros curvisetus Cleve are abundant and cosmopolitan diatoms forming bent/coiled chains of cells with curved setae. The analysis of the LSU rDNA D1-D4 region [1], and of 18S rDNA [2] showed that three lineages (*C. curvisetus* 1, 2 and 3) co-occurring in the GoN, which are probably distinct species, form a monophyletic clade including *C. pseudocurvisetus* Mangin. The latter also forms bent chains with curved setae but is distinct from *C. curvisetus* at the attachment of the setae to the valve. The number of species in the *C. curvisetus* complex could be as high as 11, including two potential new species within lineage 2 [3]. LM data show that both *C. curvisetus* and *C. pseudocurvisetus* in the GoN are abundant in winter and during the spring bloom [4].

Leptocylindrus

Species of the genus *Leptocylindrus* diverged long-time ago but show morphological stasis [5]. Morphological and molecular analyses of strains from the GoN showed that *L. danicus* var. *apora* French III & Hargraves deserved the species status as *L. aporus* (French III & Hargraves) Nanjappa & Zingone, while two novel species roughly matching the original description of *L. danicus* were described as *L. convexus* Nanjappa & Zingone and *L. hargravesii* Nanjappa & Zingone [6].
While *L. convexus* can be identified by valve shape, *L. hargravesii* differs from *L. danicus* for subtle ultrastructural characteristics, whereas *L. aporus* and *L. minimus* differ from *L. danicus* and *L. hargravesii* for both ultrastructural details and plastid number and shape. Being plastids not well appreciated in fixed material, a single taxon, *L.* cf. *danicus,* including *L. danicus*, *L. hargravesii* and *L. aporus,* is routinely identified in LM at LTER-MareChiara, while *L. convexus* is counted separately, beginning in August 2011, when it was identified as a separate species. In the GoN, these species were deemed to succeed one another throughout the year [5].

Pseudo-nitzschia spp.

Pseudo-nitzschia is a genus undergoing recent intense diversification. It comprises 62 recognized species [7] among which many cryptic taxa [8]. We focused on three species groups, namely the *P. delicatissima, P. galaxiae* and *P. pseudodelicatissima* complexes.

Variations in frustule ultrastructure among taxa previously attributed to *P. delicatissima* (Cleve) Heiden have been recognized since the 1980's (Rivera 1985 in [9]), while molecular analyses have led to the description of several different species, some of which are totally cryptic. In the GoN, *P. decipiens* Lundholm & Moestrup, *P. dolorosa* Lundholm & Moestrup, *P. arenysensis* Quijano-Scheggia, Garcés & Lundholm and *P. allochrona* Zingone, Percopo & Sarno [9,10,11] live in sympatry but with distinct phenology, in some cases: *P. allochrona* occurs in late summer, while *P. delicatissima*, *P. arenysensis* and the less abundant *P. dolorosa* co-occur in spring [12].

P. galaxiae Lundholm & Moestrup was described in 2002 from Mexican and Australian waters [13] and then reported from the GoN [14] where it occurred with three different morphotypes (small, medium, and large). These corresponded to five different but phylogenetically close LSU rDNA ribotypes, suggesting that *P. galaxiae* is a species complex [15]. However, not all V4-18S rDNA sequences of those ribotypes are known, limiting the comparison with the ASVs obtained in the present work. In the GoN,

the small morphotype is present in winter, while the medium and large morphotypes are present from spring to summer [14].

Morphological and molecular variations among strains of *P. pseudodelicatissima* (Hasle) Hasle justified the delineation of two new species, *P. caciantha* Lundholm, Moestrup & Hasle and *P. calliantha* Lundholm, Moestrup & Hasle, and highlighted differences between *P. pseudodelicatissima* and the similar *P. cuspidata* (Hasle) Hasle [16]. Since then, new species are continuously described within the *P. pseudodelicatissima* complex, including *P. mannii* Amato & Montresor and *P. hasleana* Lundholm. In the GoN, all the species mentioned here have been recorded prevalently in autumn in molecular (clone library) investigations [15].

Azadinium

The genus *Azadinium*, established with the description of *A. spinosum* Elbrächter & Tillman [17], includes small species that have likely been misidentified in the past due to the lack of distinctive features in light microscopy. The number of species is steadily increasing because of the interest raised by the ability of many of them to produce toxins (azaspiracids). Of the 17 known species [7,18], *A. dexteroporum* Percopo & Zingone was described from the GoN [19] and *A. poporum* Tillman & Elbrächter was observed at several places in the Mediterranean Sea [20,21]. A high intraspecific diversity has been discovered, e.g., in *A. poporum*, with ribotypes differing in their environmental preferences [21]. Because of identification problems in LM, information on the distribution of *Azadinium* species in the GoN is scanty and fragmentary.

Heterocapsa

The genus *Heterocapsa* includes small peridinioid dinoflagellates with a complex taxonomic history. Three species previously attributed to *Heterocapsa* were transferred to other genera, including the original type species *H. triquetra* (Ehrenb.) F.Stein, while four species originally attributed to other genera are now recognized as *Heterocapsa.* As of 2025, there are 26 recognized species of *Heterocapsa* [18], of which six has been described between 2020 and 2022 [22–25], underscoring the probability of more undescribed species of *Heterocapsa*. Recent new species were mostly described from Asian coastal waters, where their blooms can affect aquaculture production [25]. Identification in light microscopy is difficult, as the body scale ultrastructure is considered the most reliable character [26,27]. In the GoN, limited information is available on *Heterocapsa* morphotypes, mostly identified as *H. niei* (A.R. Loebl.) L.C. Morrill & A.R. Loebl., which peak in late spring-beginning of summer [28,29].

Micromonas

Micromonas species are neglected in routine LM analyses because they are small (2-3 µm) and damaged by fixation. The genus originally comprised only *M. pusilla* (Butcher) Manton & Parke, which was considered cosmopolitan and ubiquitous. Molecular phylogeny revealed several deeply diverging clades [30–32] with different ecological niches [33], suggesting the existence of cryptic species. The species *M. commoda* Baren, Bachy & Worden [34], *M. bravo*

Simon, Foulon & Marin and *M. polaris* Simon, Foulon & Marin [35] were subsequently described. Finally, candidate's species and clades within *M. bravo* and *M. commoda* were renamed by [36], resulting in a list of 9 potential species (*M. commoda* A1, *M. commoda* A2, *M. bravo* B1, *M. bravo* B2, *M. sp* B3, *M. sp* B4, *M. sp* B5, *M. pusilla*, and *M. polaris*). In the GoN, the seasonal distribution of a species identified as *M. pusilla* was described in LM studies based on serial dilution culturing [37]. This taxon was abundant in winter [38] and different strains showed variable susceptibility to viral infection [39]. Strains from the GoN were re-attributed to *M. bravo* (strains Mp1, Mp2, MpPart and MpCo) and *M. commoda* (strain Naples) by [35].

REFERENCES

1. Kooistra WHCF, Sarno D, Hernández-Becerril DU *et al.* Comparative molecular and morphological phylogenetic analyses of taxa in the Chaetocerotaceae (Bacillariophyta). *Phycologia* 2010;**49**:471–500.

2. Gaonkar CC, Piredda R, Sarno D *et al.* Species detection and delineation in the marine planktonic diatoms *Chaetoceros* and *Bacteriastrum* through metabarcoding: making biological sense of haplotype diversity. *Environ Microbiol* 2020;**22**:1917–29.

3. De Luca D, Piredda R, Sarno D *et al.* Resolving cryptic species complexes in marine protists: phylogenetic haplotype networks meet global DNA metabarcoding datasets. *ISME Journal* 2021;**15**:1931–42.

4. Longobardi L. From data to knowledge: integrating observational data to trace phytoplankton dynamics in a changing world. 2020.

5. Nanjappa D, Kooistra WHCF, Zingone A. A reappraisal of the genus *Leptocylindrus* (Bacillariophyta), with the addition of three species and the erection of *Tenuicylindrus* gen. nov. *J Phycol* 2013;**49**:917–36.

6. Nanjappa D, Audic S, Romac S *et al.* Assessment of species diversity and distribution of an ancient diatom lineage using a DNA metabarcoding approach. *PLoS One* 2014;**9**, DOI: 10.1371/journal.pone.0103810.

7. Guiry MD, Guiry GM. AlgaeBase, https://www.algaebase.org . *World-Wide Electronic Publication, University of Galway* 2025. Accessed on 14 July 2025.

8. Bates SS, Hubbard KA, Lundholm N *et al. Pseudo-nitzschia*, *Nitzschia*, and domoic acid: New research since 2011. *Harmful Algae* 2018;**79**:3–43.

9. Lundholm N, Moestrup Ø, Kotaki Y *et al.* Inter- and intraspecific variation of the *Pseudo-nitzschia delicatissima* complex (Bacillariophyceae) illustrated by rRNA probes, morphological data and phylogenetic analyses. *J Phycol* 2006;**42**:464–81.

10. Quijano-Scheggia SI, Garcés E, Lundholm N *et al.* Morphology, physiology, molecular phylogeny and sexual compatibility of the cryptic *Pseudo-nitzschia delicatissima* complex (Bacillariophyta), including the description of *P. arenysensis* sp. nov. *Phycologia* 2009;**48**:492–509.

11. Percopo I, Ruggiero MV, Sarno D *et al.* Phenological segregation suggests speciation by time in the planktonic diatom *Pseudo-nitzschia allochrona* sp. nov. *Ecol Evol* 2022;**12**, DOI: 10.1002/ece3.9155.

12. Ruggiero MV, Kooistra WHCF, Piredda R *et al.* Temporal changes of genetic structure and diversity in a marine diatom genus discovered via metabarcoding. *Environmental DNA* 2022;**4**:763–75.

13. Lundholm N, Moestrup Ø. The marine diatom *Pseudo-nitzschia galaxiae* sp. nov. (Bacillariophyceae): Morphology and phylogenetic relationships. *Phycologia* 2002;**41**:594–605.

14. Cerino F, Orsini L, Sarno D *et al.* The alternation of different morphotypes in the seasonal cycle of the toxic diatom *Pseudo-nitzschia galaxiae*. *Harmful Algae* 2005;**4**:33–48.

15. Ruggiero MV, Sarno D, Barra L *et al.* Diversity and temporal pattern of *Pseudo-nitzschia* species (Bacillariophyceae) through the molecular lens. *Harmful Algae* 2015;**42**:15–24.

16. Lundholm N, Moestrup Ø, Hasle GR *et al.* A study of the *Pseudo-nitzschia pseudodelicatissima/cuspidata* complex (Bacillariophyceae): What is *P. pseudodelicatissima*? *J Phycol* 2003;**39**:797–813.

17. Tillmann U, Elbrächter M, Krock B *et al.* *Azadinium spinosum* gen. et sp. nov. (Dinophyceae) identified as a primary producer of azaspiracid toxins. *Eur J Phycol* 2009;**44**:63–79.

18. Salas R, Tillmann U, Gu H *et al.* Morphological and molecular characterization of multiple new *Azadinium* strains revealed a high diversity of non-toxigenic species of Amphidomataceae (Dinophyceae) including two new *Azadinium* species in Irish waters, North East Atlantic. *Phycological Res* 2021;**69**:88–115.

19. Percopo I, Siano R, Rossi R *et al.* A new potentially toxic *Azadinium* species (Dinophyceae) from the Mediterranean Sea, *A. dexteroporum* sp. nov. *J Phycol* 2013;**49**:950–66.

20. Luo Z, Krock B, Mertens KN *et al.* Adding new pieces to the *Azadinium* (Dinophyceae) diversity and biogeography puzzle: Non-toxigenic *Azadinium zhuanum* sp. nov. from China, toxigenic *A. poporum* from the Mediterranean, and a non-toxigenic *A. dalianense* from the French Atlantic. *Harmful Algae* 2017;**66**:65–78.

21. Luo Z, Krock B, Giannakourou A *et al.* Sympatric occurrence of two *Azadinium poporum* ribotypes in the Eastern Mediterranean Sea. *Harmful Algae* 2018;**78**:75–85.

22. Sunesen I, Rodríguez F, Tardivo Kubis JA *et al.* Morphological and molecular characterization of *Heterocapsa claromecoensis* sp. nov. (Peridiniales, Dinophyceae) from Buenos Aires coastal waters (Argentina). *Eur J Phycol* 2020:490–506.

23. Benico G, Lum WM, Takahashi K *et al.* Thecal tabulation, body scale morphology and phylogeny of *Heterocapsa philippinensis* sp. nov. (Peridiniales, Dinophyceae) from the Philippines. *Eur J Protistol* 2021;**80**, DOI: 10.1016/j.ejop.2021.125811.

24. Choi H, Kim S. *Heterocapsa busanensis* sp. nov. (Peridiniales, Dinophyceae): A new marine thecate dinoflagellate from Korean coastal waters. *Eur J Protistol* 2021;**79**, DOI: 10.1016/j.ejop.2021.125797.

25. Hanifah AH, Teng ST, Law IK *et al.* Six marine thecate *Heterocapsa* (Dinophyceae) from Malaysia, including the description of three novel species and their cytotoxicity potential. *Harmful Algae* 2022;**120**, DOI: 10.1016/j.hal.2022.102338.

26. Iwataki M. Taxonomy and identification of the armored dinoflagellate genus *Heterocapsa* (Peridiniales, Dinophyceae). *Plankton Benthos Res* 2008;**3(3)**:135–42.

27. Gottschling M, Tillmann U, Kusber WH *et al.* A gordian knot: Nomenclature and taxonomy of *Heterocapsa triquetra* (peridiniales: Heterocapsaceae). *Taxon* 2018;**67**:179–85.

28. Mordret S, Piredda R, Vaulot D *et al.* DINOREF: A curated dinoflagellate (Dinophyceae) reference database for the 18S rRNA gene. *Mol Ecol Resour* 2018;**18**:974–87.

29. Zingone A, Tortora C, D'Alelio D *et al.* Assembly rules vary seasonally in stable phytoplankton associations of the Gulf of Naples (Mediterranean Sea). *Marine Ecology* 2022;**44**, DOI: 10.1111/maec.12730.

30. Šlapeta J, López-García P, Moreira D. Global dispersal and ancient cryptic species in the smallest marine eukaryotes. *Mol Biol Evol* 2006;**23**:23–9.

31. Guillou L, Eikrem W, Chrétiennot-Dinet M-J *et al.* Diversity of picoplanktonic prasinophytes assessed by direct nuclear SSU rDNA sequencing of environmental samples and novel isolates retrieved from oceanic and coastal marine ecosystems. *Protist* 2004;**155**:193–214.

32. Worden AZ, Lee J-H, Mock T *et al.* Green evolution and dynamic adaptations revealed by genomes of the marine picoeukaryotes *Micromonas*. *Science (1979)* 2009;**324**:268–72.

33. Foulon E, Not F, Jalabert F *et al.* Ecological niche partitioning in the picoplanktonic green alga *Micromonas pusilla*: Evidence from environmental surveys using phylogenetic probes. *Environ Microbiol* 2008;**10**:2433–43.

34. van Baren MJ, Bachy C, Reistetter EN *et al.* Evidence-based green algal genomics reveals marine diversity and ancestral characteristics of land plants. *BMC Genomics* 2016;**17**, DOI: 10.1186/s12864-016-2585-6.

35. Simon N, Foulon E, Grulois D *et al.* Revision of the genus *Micromonas* Manton et Parke (Chlorophyta, Mamiellophyceae), of the type species *M. pusilla* (Butcher) Manton & Parke and of the species *M. commoda* van Baren, Bachy and Worden and description of two new species based on the genetic and phenotypic characterization of cultured isolates. *Protist* 2017;**168**:612–35.

36. Tragin M, Vaulot D. Novel diversity within marine Mamiellophyceae (Chlorophyta) unveiled by metabarcoding. *Sci Rep* 2019;**9**, DOI: 10.1038/s41598-019-41680-6.

37. Throndsen J, Zingone A. Micronomads of the Mediterranean sea. *Giornale botanico italiano* 1994;**128**:1029–106.

38. Zingone A, Sarno D, Forlani G. Seasonal dynamics in the abundance of *Micromonas pusilla* (Prasinophyceae) and its viruses in the Gulf of Naples (Mediterranean Sea). *J Plankton Res* 1999;**21**:2143–59.

39. Zingone A, Natale F, Biffali E *et al.* Diversity in morphology, infectivity, molecular characteristics and induced host resistance between two viruses infecting *Micromonas pusilla*. *Aquatic Microbial Ecology* 2006;**45**:1–14.
