# Supplementary material for: Niche divergence suggests ecophysiological differences between cryptic phytoplankton species
Source: ISME Commun. 2026 Aug 4;6(1):ycag225. doi: 10.1093/ismeco/ycag225 (PMC13590887; doi:10.1093/ismeco/ycag225)
Supplement: Supplementary file 1 [file web_material_ycag225.zip › Supplementary tables_1-4_ycag225.pdf]

Table S1 Niche parameters of cryptic taxa in the Outlying Mean Index (OMI) analysis, expressed in absolute and relative values. Marginality indicates the distance between the average environmental conditions occupied by the species and the average environmental conditions of all samples. P values (n.s.: non-significant, * p <0.05, ** p < 0.01, *** p < 0.001) indicate the significance of this distance, with non-significant distances indicating no preferences for particular environmental conditions in the space analysed. Tolerance is equivalent to niche breadth. Residual tolerance is the variance of the species niche not represented on the bi-dimensional plane of the OMI.

Species group	Species	Marginality	Tolerance	Residual tolerance	Relative marginality (%)	Relative tolerance (%)	Relative residual tolerance (%)
Chaetoceros curvisetus	*C. curvisetus* 1	1.65***	0.99	5.91	19.4	11.6	69.0
	C. curvisetus 2b	0.30***	1.70	5.19	4.3	23.7	72.0
	C. curvisetus 3	0.38n.s.	1.07	6.42	4.9	13.6	81.5
	C. pseudocurvisetus	0.98***	1.80	4.72	13.2	24.0	62.8
Leptocylindrus	*L. aporus*	0.32***	2.22	3.88	5.0	34.6	60.4
	L. convexus	0.60***	1.46	4.72	8.9	21.6	69.5
	L. danicus	0.14**	1.98	4.56	2.1	29.7	68.2
	L. hargravesii	1.18***	1.93	3.27	18.6	30.3	51.2
Pseudo-nitzschia delicatissima	*P. allochrona*	2.09***	0.85	2.48	38.6	15.7	45.6
	P. arenysensis/dolorosa	1.63***	0.69	4.99	22.3	9.6	68.1
	P. delicatissima	1.32***	1.06	4.85	18.3	14.7	67.0
Pseudo-nitzschia galaxiae	*P. galaxiae* B571	3.06***	1.19	6.82	27.6	10.8	61.6
	P. galaxiae B606	0.17n.s.	0.82	5.91	2.6	11.9	85.5
	P. galaxiae B617	1.52***	1.02	3.05	27.1	18.3	54.5
Pseudo-nitzschia pseudodelicatissima	*P. caciantha/linea*	0.97n.s.	0.54	4.66	15.8	8.8	75.4
	P. calliantha	0.62n.s.	2.63	2.51	10.8	45.6	43.6
	P. mannii	1.97*	1.83	1.49	37.2	34.7	28.1
	P. pseudodelicatissima	1.40*	3.15	4.88	14.9	33.4	51.7
Azadinium	*A. concinnum/caudatum*	1.52n.s.	1.34	4.45	20.8	18.4	60.8
	A. dexteroporum	1.96**	0.47	3.37	33.8	8.1	58.0

	Azadinium sp.	1.75***	0.63	4.24	26.4	9.5	64.1
Heterocapsa	*H. horiguchi*	2.94***	1.19	3.79	37.1	15.1	47.8
	H. pygmea	2.58**	0.90	3.62	36.3	12.7	51.0
	H. limii	1.98***	0.74	4.31	28.2	10.6	61.2
	H. niei/iwataki	1.96***	1.11	4.67	25.3	14.4	60.3
	H. rotundata	3.10***	3.32	6.13	24.7	26.5	48.8
	Heteropasa sp.	0.83***	1.43	4.78	11.8	20.3	67.8
Micromonas	*M. bravo* B1	1.80***	1.49	5.88	19.7	16.2	64.1
	M. bravo B2	1.44**	1.97	4.35	18.5	25.4	56.0
	M. commoda A1	2.15**	1.87	5.19	23.4	20.3	56.3
	M. commoda A2	3.47**	2.17	8.46	24.6	15.4	60.0
	M. clade B4	2.22***	1.55	4.69	26.3	18.3	55.4
	M. clade B5	1.49***	0.46	2.18	36.1	11.2	52.8

Table S2 Preferential distribution of cryptic taxa during the year (Julian days) and along gradients of environmental variables. Values refer to the interquartile range (IQR), i.e, the 25th-75th percentile interval of the distribution of read abundance and of the corresponding environmental variables.

Species group	Species	Time (Julian day)	Temperature (°C)	Salinity	MLD (m)	Day length (h)	NO3 (mmol m^{-3})	PO4 (mmol m^{-3})	Si(OH)4 (mmol m^{-3})
Chaetoceros curvisetus	*C. curvisetus* 1	51-75	13.90-14.96	37.60-38.14	17.31-42.00	10.73-11.97	0.30-2.15	0.02-0.09	0.35-2.86
	C. curvisetus 2b	132-210	19.02-28.41	37.09-37.82	10.15-10.89	13.40-14.55	0.06-0.93	0.06-0.09	0.16-1.34
	C. curvisetus 3	79-295	15.68-22.69	37.64-37.85	14.22-18.64	10.83-12.03	0.56-1.59	0.02-0.05	0.91-2.00
	C. pseudocurvisetus	322-45	14.55-19.00	37.60-37.99	13.48-33.24	9.88-11.03	0.64-3.73	0.03-0.11	1.45-4.84
Leptocylindrus	*L. aporus*	210-239	24.48-28.00	37.74-37.95	10.15-10.39	13.37-14.49	0.06-0.27	0.03-0.09	0.36-1.13
	L. convexus	162-190	23.48-24.31	37.34-37.52	10.24-10.34	14.41-15.15	0.14-0.51	0.02-0.09	1.22-2.54
	L. danicus	125-289	17.59-22.37	37.19-37.81	10.52-15.45	11.16-14.59	0.19-1.27	0.05-0.09	0.81-2.20
	L. hargravesii	197-253	24.48-26.81	37.42-37.95	10.18-10.63	12.50-14.41	0.10-0.45	0.02-0.09	0.56-1.43
Pseudo-nitzschia delicatissima	*P. allochrona*	194-253	25.23-28.22	37.26-37.80	10.27-10.47	12.74-14.93	0.09-0.71	0.02-0.05	0.26-1.26
	P. arenysensis/dolorosa	73-109	13.90-16.57	37.19-37.76	11.23-17.89	11.91-13.50	0.93-2.06	0.04-0.14	0.81-3.10
	P. delicatissima	76-133	14.35-17.67	37.08-37.60	10.57-17.31	12.03-14.44	0.32-1.58	0.04-0.14	0.81-1.86
Pseudo-nitzschia galaxiae	*P. galaxiae* B571	45-72	13.85-14.57	37.76-38.00	15.55-66.66	10.68-11.83	0.30-1.61	0.03-0.08	1.12-3.76
	P. galaxiae B606	83-138	14.66-19.6	37.22-37.60	10.56-17.31	12.03-14.36	0.15-1.61	0.04-0.09	0.81-2.20
	P. galaxiae B617	185-249	23.95-27.87	37.31-37.84	10.15-10.60	12.94-14.97	0.09-0.30	0.01-0.12	0.49-1.04
Pseudo-nitzschia pseudodelicatissima	*P. caciantha/linea*	73-97	14.41-14.58	37.43-37.87	11.57-32.94	10.58-12.55	0.30-2.58	0.02-0.08	0.39-2.44
	P. calliantha	199-213	22.22-25.75	37.42-37.71	10.27-10.32	13.68-14.78	0.01-0.31	0.00-0.03	0.26-1.26
	P. mannii	200-207	22.22-25.75	37.57-37.75	10.17-10.32	13.25-14.78	0.00-0.18	0.00-0.03	0.24-0.52
	P. pseudodelicatissima	76-103	13.90-16.73	37.19-37.60	11.69-17.31	12.03-13.24	1.58-5.28	0.04-0.20	0.81-6.16
Azadinium	*A. concinnum/caudatum*	134-157	18.12-21.80	37.00-37.09	10.36-10.58	14.47-15.04	0.00-2.93	0.05-0.20	0.42-5.96
	A. dexteroporum	110-161	16.06-22.88	36.96-37.51	10.29-11.23	13.56-15.10	0.02-2.13	0.02-0.10	0.54-2.90
	Azadinium sp.	106-167	16.52-23.70	36.96-37.40	10.25-10.62	13.39-15.06	0.02-1.19	0.04-0.12	1.00-2.85
Heterocapsa	*H. horiguchi*	79-173	13.97-23.42	36.77-37.51	10.28-15.55	11.18-14.80	0.01-4.21	0.10-0.19	1.74-3.90
	H. pygmea	136-173	19.91-25.30	36.77-37.36	10.23-10.61	13.98-15.10	0.05-2.13	0.04-0.10	0.64-5.84
	H. limii	155-258	20.25-25.93	36.99-37.80	10.21-10.94	12.44-14.87	0.08-2.13	0.04-0.12	0.64-4.16
	H. niei/iwataki	133-171	19.21-24.31	36.76-37.43	10.21-10.65	14.44-15.13	0.43-2.13	0.06-0.17	0.64-4.31
	H. rotundata	48-87	14.01-14.96	37.32-37.81	13.48-45.71	10.58-11.83	1.26-5.68	0.07-0.12	1.76-9.26
	Heterocapsa sp.	131-171	18.84-23.15	36.77-37.14	10.21-10.36	13.98-15.10	0.09-2.91	0.04-0.12	0.64-2.85

Micromonas	*M. bravo* B1	43-72	14.01-15.20	37.71-37.99	15.55-55.00	10.02-11.62	0.64-2.58	0.03-0.09	1.46-3.76
	M. bravo B2	341-43	14.91-18.77	37.60-37.98	15.76-47.69	9.408-10.78	0.68-2.58	0.03-0.09	1.41-3.76
	M. commoda A1	43-73	13.93-14.91	37.82-38.00	28.57-66.66	10.58-11.91	0.30-2.58	0.03-0.07	1.12-2.44
	M. commoda A2	67-73	14.02-14.58	37.61-38.14	17.32-33.24	11.63-11.91	0.30-2.58	0.02-0.08	0.24-2.44
	M. clade B4	341-43	14.58-18.07	37.62-38.00	18.10-50.57	9.41-10.78	0.56-2.58	0.03-0.09	1.27-4.20
	M. clade B5	257-259	24.48-25.23	37.74-38.08	10.34-14.22	12.49-12.60	0.46-1.44	0.02-0.07	1.43-1.95

Table S3 Seasonal overlap (SO), co-occurrence ratio (CoOC) and niche overlap (NO) of the cryptic taxa pairs. SO is the overlap between the KDE curves representing the seasonal distribution of two taxa. CoOC is the proportion of samples with both taxa to the total number of samples with one or both of them. NO is the average across NOs for environmental variables (NO-salinity, NO-MLD, etc.), calculated as the overlap between the KDE areas of the distribution of the taxa pairs along each variable (Fig. 1).

Species complex	Species 1	Species 2	SO	CoOC	NO mean	NO-temperature	NO-salinity	NO-MLD	NO-nutrient	NO-day length
Chaetoceros curvisetus	*C. curvisetus* 1	*C. curvisetus* 2b	0.20	0.33	0.38	0.27	0.59	0.26	0.55	0.24
	C. curvisetus 1	*C. curvisetus* 3	0.32	0.16	0.51	0.34	0.60	0.54	0.57	0.50
	C. curvisetus 1	*C. pseudocurvisetus*	0.52	0.50	0.68	0.62	0.75	0.81	0.67	0.54
	C. curvisetus 2b	*C. curvisetus* 3	0.30	0.17	0.52	0.61	0.64	0.37	0.69	0.29
	C. curvisetus 2b	*C. pseudocurvisetus*	0.23	0.36	0.44	0.50	0.66	0.32	0.49	0.23
	C. curvisetus 3	*C. pseudocurvisetus*	0.48	0.21	0.62	0.59	0.76	0.67	0.52	0.57
Leptocylindrus	*L. aporus*	*L. convexus*	0.33	0.67	0.55	0.47	0.36	0.88	0.60	0.45
	L. aporus	*L. danicus*	0.27	0.70	0.54	0.27	0.52	0.65	0.55	0.71
	L. aporus	*L. hargravesii*	0.65	0.26	0.68	0.74	0.59	0.85	0.57	0.63
	L. convexus	*L. danicus*	0.37	0.57	0.55	0.36	0.55	0.67	0.61	0.54
	L. convexus	*L. hargravesii*	0.38	0.31	0.56	0.51	0.48	0.86	0.50	0.45
	L. danicus	*L. hargravesii*	0.22	0.10	0.49	0.27	0.49	0.68	0.49	0.53
Pseudo-nitzschia delicatissima	*P. allochrona*	*P. arenysensis/dolorosa*	0.01	0.00	0.43	0.02	0.70	0.58	0.44	0.41
	P. allochrona	*P. delicatissima*	0.13	0.02	0.51	0.18	0.58	0.74	0.48	0.56
	P. arenysensis/dolorosa	*P. delicatissima*	0.78	0.63	0.80	0.80	0.80	0.81	0.81	0.77
Pseudo-nitzschia galaxiae	*P. galaxiae* B571	*P. galaxiae* B606	0.36	0.09	0.46	0.48	0.34	0.58	0.53	0.39
	P. galaxiae B571	*P. galaxiae* B617	0.04	0.06	0.28	0.05	0.42	0.24	0.55	0.13
	P. galaxiae B606	*P. galaxiae* B617	0.21	0.22	0.56	0.22	0.67	0.63	0.66	0.60
Pseudo-nitzschia pseudodelicatissima	*P. caciantha/linea*	*P. calliantha*	0.25	0.05	0.49	0.32	0.55	0.51	0.70	0.35
	P. caciantha/linea	*P. mannii*	0.15	0.08	0.42	0.15	0.49	0.57	0.55	0.34
	P. caciantha/linea	*P. pseudodelicatissima*	0.73	0.07	0.63	0.76	0.51	0.58	0.60	0.68
	P. calliantha	*P. mannii*	0.87	0.10	0.87	0.82	0.88	0.93	0.85	0.88
	P. calliantha	*P. pseudodelicatissima*	0.28	0.12	0.53	0.37	0.61	0.74	0.47	0.46
	P. mannii	*P. pseudodelicatissima*	0.18	0.14	0.46	0.20	0.57	0.76	0.34	0.45
Azadinium	*A. concinnum/caudatum*	*A. dexteroporum*	0.73	0.22	0.71	0.68	0.53	0.89	0.67	0.76

	A. concinnum/caudatum	*Azadinium* sp.	0.70	0.12	0.71	0.68	0.59	0.92	0.62	0.75
	A. dexteroporum	*Azadinium* sp.	0.82	0.32	0.81	0.85	0.74	0.92	0.72	0.83
Heterocapsa	*H. limii*	*H. niei/iwataki*	0.60	0.53	0.75	0.68	0.66	0.99	0.79	0.62
	H. limii	*H. pygmaea*	0.74	0.16	0.77	0.68	0.69	0.94	0.76	0.79
	H. limii	*H. rotundata*	0.18	0.04	0.37	0.22	0.56	0.33	0.55	0.18
	H. limii	*Heterocapsa* sp.	0.66	0.63	0.73	0.72	0.69	0.79	0.80	0.65
	H. limii	*H. horiguchii*	0.80	0.32	0.77	0.76	0.66	0.96	0.62	0.85
	H. niei/iwataki	*H. pygmaea*	0.47	0.19	0.63	0.53	0.45	0.94	0.62	0.62
	H. niei/iwataki	*H. rotundata*	0.24	0.01	0.44	0.25	0.73	0.33	0.49	0.39
	H. niei/iwataki	*Heterocapsa* sp.	0.64	0.47	0.69	0.58	0.63	0.80	0.68	0.78
	H. niei/iwataki	*H. horiguchii*	0.46	0.29	0.63	0.60	0.49	0.97	0.56	0.55
	H. pygmaea	*H. rotundata*	0.18	0.06	0.34	0.26	0.37	0.37	0.46	0.22
	H. pygmaea	*Heterocapsa* sp.	0.60	0.13	0.64	0.59	0.49	0.85	0.64	0.62
	H. pygmaea	*H. horiguchii*	0.78	0.23	0.67	0.58	0.62	0.97	0.46	0.74
	H. rotundata	*Heterocapsa* sp.	0.43	0.07	0.52	0.47	0.57	0.39	0.65	0.49
	H. rotundata	*H. horiguchii*	0.17	0.02	0.35	0.22	0.36	0.36	0.62	0.19
	Heterocapsa sp.	*H. horiguchii*	0.60	0.25	0.72	0.69	0.64	0.83	0.79	0.64
Micromonas	*M. bravo* B1	*M. clade* B4	0.58	0.51	0.72	0.60	0.80	0.84	0.74	0.63
	M. bravo B1	*M. clade* B5	0.04	0.05	0.37	0.04	0.54	0.46	0.56	0.22
	M. bravo B1	*M. commoda* A1	0.90	0.34	0.82	0.92	0.80	0.81	0.74	0.81
	M. bravo B1	*M. commoda* A2	0.63	0.18	0.63	0.81	0.56	0.72	0.60	0.45
	M. bravo B1	*M. bravo* B2	0.54	0.47	0.72	0.55	0.84	0.78	0.78	0.65
	M. clade B4	*M. clade* B5	0.16	0.19	0.40	0.13	0.59	0.41	0.67	0.21
	M. clade B4	*M. commoda* A1	0.54	0.30	0.67	0.55	0.78	0.73	0.77	0.52
	M. clade B4	*M. commoda* A2	0.36	0.15	0.51	0.41	0.59	0.65	0.57	0.34
	M. clade B4	*M. bravo* B2	0.93	0.65	0.89	0.92	0.89	0.87	0.87	0.89
	M. clade B5	*M. commoda* A1	0.05	0.05	0.37	0.05	0.59	0.32	0.68	0.20
	M. clade B5	*M. commoda* A2	0.02	0.00	0.35	0.02	0.67	0.39	0.50	0.17
	M. clade B5	*M. bravo* B2	0.20	0.19	0.40	0.17	0.50	0.46	0.63	0.22
	M. commoda A1	*M. bravo* B2	0.50	0.25	0.63	0.51	0.77	0.62	0.74	0.52
	M. commoda A1	*M. commoda* A2	0.71	0.32	0.68	0.86	0.59	0.68	0.68	0.57
	M. commoda A2	*M. bravo* B2	0.31	0.11	0.46	0.38	0.53	0.60	0.50	0.29

Table S4 Seasonal overlap (SoLM) and co-observation rate (CoOB) between ASVs detected by metabarcoding and corresponding morphs in Light Microscopy (LM). SoLM was calculated as the overlap between the KDE curves representing the seasonal distribution of each taxon from metabarcoding and LM data, respectively. CoOB is the ratio of samples in which one taxon was detected by both methods over the number of samples where it was detected by one or both methods. 0 indicates no co-observation by the two methods, 1 indicates co-observation on all sampling dates.

Species complex	Metabarcoding	LM	Seasonal overlap (SoLM)	Co-observation rate (CoOB)
Chaetoceros curvisetus	*C. curvisetus* 1	*C. curvisetus*	0.24	0.47
	C. curvisetus 2b	*C. curvisetus*	0.69	0.60
	C. curvisetus 3	*C. curvisetus*	0.39	0.18
	C. pseudocurvisetus	*C. pseudocurvisetus*	0.34	0.51
Leptocylindrus	*L. aporus*	*L.* cf. *danicus*	0.59	0.93
	L. danicus	*L.* cf. *danicus*	0.47	0.70
	L. hargravesii	*L.* cf. *danicus*	0.60	0.24
	L. convexus	*L. convexus*	0.82	0.57
Pseudo-nitzschia delicatissima	*P. allochrona*	*P. delicatissima*	0.56	0.34
	P. arenysensis/dolorosa	*P. delicatissima*	0.38	0.26
	P. delicatissima	*P. delicatissima*	0.51	0.30
Pseudo-nitzschia galaxiae	*P. galaxiae* B571	*P. galaxiae* small morph	0.38	0.43
	P. galaxiae B571	*P. galaxiae* large morph	0.14	0.10
	P. galaxiae B606	*P. galaxiae* small morph	0.69	0.29
	P. galaxiae B606	*P. galaxiae* large morph	0.62	0.37
	P. galaxiae B617	*P. galaxiae* small morph	0.11	0.17
	P. galaxiae B617	*P. galaxiae* large morph	0.55	0.47
Pseudo-nitzschia pseudodelicatissima	*P. caciantha/linea*	*P. linea*	0.60	0.24
	P. caciantha/linea	*P. pseudodelicatissima*	0.48	0.09
	P. calliantha	*P. pseudodelicatissima*	0.45	0.14
	P. mannii	*P. pseudodelicatissima*	0.35	0.07
	P. pseudodelicatissima	*P. pseudodelicatissima*	0.57	0.12
Azadinium	*A. concinnum/caudatum*	*Azadinium* sp.		0.07
	A. dexteroporum	*Azadinium* sp.		0.00
	Azadinium sp.	*Azadinium* sp.		0.00
Heterocapsa	*H. horiguchi*	*H. minima/rotundata*	0.56	0.07

	H. horiguchi	*H. niei*	0.76	0.18
	H. pygmea	*H. minima/rotundata*	0.38	0.05
	H. pygmea	*H. niei*	0.71	0.07
	H. limii	*H. minima/rotundata*	0.42	0.11
	H. limii	*H. niei*	0.79	0.21
	H. niei/iwataki	*H. minima/rotundata*	0.24	0.00
	H. niei/iwataki	*H. niei*	0.45	0.14
	H. rotundata	*H. minima/rotundata*	0.12	0.11
	H. rotundata	*H. niei*	0.10	0.00
	Heterocapsa sp.	*H. minima/rotundata*	0.39	0.12
	Heterocapsa sp.	*H. niei*	0.57	0.17
